# Engineered Melittin Delivers a Drug-Loaded ‘Chemo-Sting’ to Overcome Efflux-Mediated Multidrug Resistance in Cancer Cells

**DOI:** 10.3390/pharmaceutics18070853

**Published:** 2026-07-14

**Authors:** Nurul Ain Mohammad Hamdi, Aya M. Emam, Richard A. Bryce, Constantinos Demonacos, Jian R. Lu, Harmesh S. Aojula

**Affiliations:** 1 Division of Pharmacy and Optometry, School of Health Sciences, Faculty of Biology, Medicine, and Health, University of Manchester, Oxford Road, Manchester M13 9PT, UK; nurulain.bintimohammadhamdi@postgrad.manchester.ac.uk (N.A.M.H.); aya.emam@manchester.ac.uk (A.M.E.); richard.bryce@manchester.ac.uk (R.A.B.); constantinos.demonacos@manchester.ac.uk (C.D.); 2 Department of Medicinal Chemistry, Faculty of Pharmacy, Zagazig University, Zagazig 44519, Egypt; 3 Biological Physics, Department of Physics and Astronomy, University of Manchester, Manchester M13 9PL, UK; j.lu@manchester.ac.uk

**Keywords:** melittin analogue, membrane-active peptide, reversible drug binding, doxorubicin, multidrug resistance, molecular dynamics simulations

## Abstract

**Background:** Multidrug efflux proteins, frequently overexpressed in cancer cells, reduce intracellular drug accumulation and limit chemotherapeutic efficacy. Short drug-binding peptides containing the WXXW motif have been shown to non-covalently bind multidrug resistance (MDR)-associated drugs, potentially masking structural features recognized by efflux transporters. We hypothesized that incorporating this motif into a membrane-active peptide would generate a hybrid analogue capable of reversible drug binding and enhanced cellular uptake. **Methods:** A melittin-derived peptide (M3) was rationally designed by introducing the WXXW motif into the flexible region adjacent to the conserved proline kink to generate a dual-functional peptide with membrane activity and reversible drug binding. Membrane activity was evaluated using liposome leakage assays, and drug-binding interactions with doxorubicin were characterized using fluorescence quenching and microscale thermophoresis (MST). Molecular dynamics simulations were performed to elucidate binding mechanisms, and functional effects were assessed using calcein AM efflux assays, confocal imaging, and cytotoxicity studies across cancer cell lines. **Results:** M3 retained membrane activity and exhibited moderate, reversible binding to doxorubicin, with simulations showing binding initiation at the WXXW motif and extension to tryptophan residues W12, W15, and W19, forming a multivalent aromatic interface that suggested shielding of key drug functionalities. Functionally, M3 enhanced intracellular calcein retention and increased doxorubicin accumulation, and combination treatment produced synergistic cytotoxicity in the multidrug-resistant H69AR cell line with reduced toxicity toward normal epithelial cells. **Conclusions:** M3 acts as a membrane-active, reversible drug-binding peptide that enhances intracellular drug accumulation, supporting its potential as a modular strategy to overcome efflux-mediated MDR.

## 1. Introduction

Cancer remains a major global health challenge, with close to 20 million new cases and 9.7 million deaths reported in 2022. Breast, lung, and colorectal cancers account for over 30% of diagnosed cases [1]. Despite progress in treatment modalities such as surgery, radiation, and monoclonal antibody therapies, conventional chemotherapy remains a cornerstone for managing advanced-stage cancers [2,3]. However, their effectiveness is frequently undermined by multidrug resistance (MDR), a leading cause of cancer relapse and treatment failure, accounting for 90% of cancer-related deaths [4,5]. A primary mechanism of MDR is the overexpression of P-glycoprotein (Pgp) and multidrug resistance protein 1 (MRP1) efflux pumps that actively expel various chemotherapeutic agents, reducing their intracellular concentration and therapeutic efficacy [6]. Although MDR inhibitors have been explored as chemosensitizers, their high toxicity and limited clinical benefits pose significant challenges [7].

Short peptides containing the WXXW motif, discovered by Popkov, offer a novel approach in evading Pgp-mediated efflux [8,9]. These drug-binding peptides mimic Pgp’s hydrophobic domain and non-covalently bind to anticancer drugs, shielding them from Pgp recognition. The WXXW motif, where ‘W’ represents tryptophan and ‘X’ denotes any amino acid, forms a hydrophobic pocket facilitated by the tryptophan residues [9]. This hydrophobic pocket stabilizes the drug-peptide complex primarily through non-covalent interactions, involving π-π stacking interactions, as confirmed by molecular dynamics (MD) simulations [10].

Moreover, conventional anticancer drugs struggle to reach tumour sites due to physiological barriers, complex tumour structures, and altered microenvironments [11]. Membrane-active peptides can transport anticancer agents across lipid membranes through mechanisms such as disruption, translocation, or fusion [12]. Melittin, a membrane-active peptide derived from European honeybee venom (*Apis mellifera*), is a 26-residue amphiphilic peptide capable of disrupting cell membranes. In addition to its membrane activity, melittin has potent anticancer effects against various cancers [13]. Its dual role as a membrane-active and anticancer agent makes it a promising candidate for cancer therapy and drug delivery.

In this study, we aimed to develop a hybrid membrane-active peptide with reversible drug-binding capability to overcome multidrug resistance in cancer. By incorporating the WXXW drug-binding motif into the core region of melittin, we hypothesized that the resulting analogue would retain its membrane-disrupting and anticancer properties while enabling reversible binding with doxorubicin. In this study, melittin-derived M3 peptide was assessed for membrane activity, drug-binding affinity, efflux evasion, intracellular drug accumulation, and cytotoxicity in resistant cancer cell models. These findings support a multifunctional peptide design to enhance chemotherapeutic efficacy in MDR cancer cells.

## 2. Materials and Methods

### 2.1. Materials

Boc-protected amino acids, MBHA resin, HBTU, DIPEA, triisopropylsilane (TIS), and the Kaiser test reagents (ninhydrin and pyridine) were obtained from Novabiochem (Beeston, UK) and Sigma-Aldrich (Poole, UK). TFA, DMF, and organic solvents including DCM, methanol, ethanol, toluene, diethyl ether, and acetic acid were purchased from Fluorochem Ltd. (Old Glossop, UK) and Fisher Scientific (Leicestershire, UK). PBS, sodium acetate buffer components, glycine-HCl, ethanolamine-HCl, Triton X-100, Tween-20, DMSO, cy5-NHS, calcein AM, Hoechst 33342, BSA, antifade mounting medium, and formalin (3.7%) were obtained from Sigma-Aldrich or Fisher Scientific. Doxorubicin hydrochloride and cytarabine were purchased from Cayman Chemical (Ann Arbor, MI, USA). H69 and H69AR small-cell lung cancer cell lines, as well as A2780 and A2780cis ovarian cancer cell lines, were obtained from Caltag Medsystems (Buckingham, UK). The breast cancer cell lines MCF-7 and MDA-MB-231 were kindly provided by Dr Costas Demonacos (School of Health Sciences, Faculty of Biology, Medicine and Health, University of Manchester, UK). DMEM, DPBS, fetal bovine serum (FBS), penicillin–streptomycin, and TrypLE Express were from Gibco (Paisley, UK). CellTiter-Blue reagent was purchased from Promega (Madison, WI, USA). Primary antibodies against Pgp and MRP1 were obtained from Abcam (Cambridge, UK), and Alexa Fluor 488–conjugated secondary antibody and DAPI were from Thermo Fisher Scientific (Waltham, MA, USA). The melittin structure (PDB ID: 2MLT) was downloaded from the RCSB Protein Data Bank, and the chemical structure of doxorubicin (CID: 31703) from PubChem. Melittin was purchased from Cambridge Bioscience Limited (Cambridge, UK).

### 2.2. Methods

#### 2.2.1. Peptide Design

The hybrid melittin analogue M3 was rationally designed using melittin as the base sequence [14]. A tryptophan-based drug-binding motif (WXXW), was incorporated to enable non-covalent interaction with doxorubicin [9].

#### 2.2.2. Physicochemical Properties Prediction

The physicochemical properties of melittin and M3 were predicted using the ProtParam tool available on the ExPASy server [15]. Parameters calculated included molecular weight, isoelectric point (pI), net charge at pH 7.4, and Grand Average of Hydropathy (GRAVY) score. Helical wheel projections and hydrophobic moment calculations were performed using the HeliQuest web server to assess amphiphilicity of a-helical peptide [16].

#### 2.2.3. Peptide Synthesis and Purification

M3 was synthesized using Boc-based solid-phase peptide synthesis (SPPS) on MBHA resin in accordance with our previously reported method for melittin [14,17]. In contrast, the Popkov A4 was synthesized separately using Fmoc-based SPPS [18]. Purification was performed using preparative RP-HPLC with a C8 column (250 mm length × 21.2 mm internal diameter). The mobile phases consisted of solvent A (water with 0.1% *v*/*v* TFA) and solvent B (acetonitrile with 0.1% *v*/*v* TFA). A maximum of 30 mg crude peptide per run (2 mL in 70% acetic acid) was loaded. Peptides were purified using optimized gradients (85–45% solvent A) over 40 min at a flow rate of 8 mL/min with detection at 228 nm. The major peak corresponding to the desired product was collected and lyophilized. Final products were characterized to show HPLC purity > 95% and M + H+ values to be within 0.5 unit of the calculated mass as measured by mass spectrometry.

#### 2.2.4. Liposome Leakage Assay

Liposome leakage assay was used to evaluate the membrane-disruptive activity of the peptides [19]. Calcein-loaded liposomes were prepared as described previously [14] and adjusted to a lipid concentration of 7.5 mg/mL. A 10 µL aliquot was taken into 2 mL of PBS (pH 7.4), and calcein release was monitored by measuring fluorescence emission at 520 nm with an excitation wavelength of 490 nm. After a 2 min baseline stabilization, 10 µL of peptide solution was added, and fluorescence was recorded for 3 min. Triton X-100 (1% *v*/*v*) was then added to achieve complete lysis, providing the maximum fluorescence value (100% leakage). Liposome leakage (%) was calculated using the following formula:

% Liposome leakage = [(F1 − F0)/(F2 − F0)] × 100(1)

where F1 is the Fluorescence intensity after addition of peptide, F0 is the initial fluorescence intensity, and F2 is the fluorescence intensity after the addition of triton-X.

#### 2.2.5. Circular Dichroism (CD)

Far-UV CD spectroscopy was used to assess peptide secondary structure [20]. Spectra were recorded in water and 50% TFE to compare solution structure with a membrane-mimetic environment. Measurements were acquired on a Chirascan V100 spectropolarimeter (Applied Photophysics, Leatherhead, UK) at room temperature using a 0.1 mm quartz cuvette, scanning from 190–260 nm with a 1 nm bandwidth and step size and 0.5 s time-per-point. Peptides (50 µM) were scanned in triplicate, and averaged spectra were corrected by subtracting buffer baselines. Ellipticity values (mdeg) were converted to mean residue ellipticity (deg·cm^2^·dmol^−1^) using the instrument software. Secondary structure content was estimated using the BestSel server [21].

#### 2.2.6. Tryptophan Quenching Assay

Fluorescence measurements were performed at room temperature using a PerkinElmer LS 55 spectrofluorometer (PerkinElmer, Waltham, MA, USA) to evaluate doxorubicin binding to M3. Samples were excited at 280 nm, and the emission spectrum was recorded from 290–450 nm with 5 nm slit widths. M3 was prepared at 1 µM in PBS (pH 7.4), and doxorubicin (0.5–250 µM) was titrated to assess quenching. Fluorescence intensities at the emission maximum were used to construct Stern–Volmer plots. Data were fitted to the modified Stern–Volmer equation [22]:

(2) $$\frac{F0}{F}=\;(1\;+\;\mathrm{Ksv}\left[Q\right])\cdot e^{\mathrm{Ka}[Q]}$$

where F0 and F are the fluorescence intensities in the absence and presence of quencher, respectively. [Q] is the doxorubicin concentration, Ksv is the Stern–Volmer quenching constant, and Ka represents the association (binding) constant.

#### 2.2.7. Microscale Thermophoresis (MST)

MST was used to determine the binding affinity between Cy5-labeled M3 (50 nM) peptide and the ligands doxorubicin and cytarabine. To do this, M3 was labeled with Cy5 dye by reacting 1 mg M3 with 1 mg Cy5-NHS ester. The dye was first dissolved in 1 mL DMSO and then mixed with 1 mL PBS (final 50% PBS/50% DMSO), and the mixture was incubated overnight at room temperature. The conjugate was purified on a preparative RP-HPLC column, and the single fluorescent peak eluting later than the free peptide was collected and its identity confirmed by mass spectrometry. Drug stock solutions were prepared in water containing 0.05% (*v*/*v*) Tween-20 to minimize nonspecific adsorption, while the peptide was dissolved in PBS supplemented with 5% (*v*/*v*) DMSO and 0.05% (*v*/*v*) Tween-20. Doxorubicin was prepared as a 2 mM stock solution and diluted as a two-fold serial dilution series. Each ligand dilution was mixed 1:1 with a constant concentration of Cy5-labeled peptide prior to loading into Monolith NT.115 Premium Capillaries. MST measurements were performed at 25 °C using a Monolith NT.115 instrument (NanoTemper Technologies, Munich, Germany) with Pico-RED excitation (5% LED power, medium MST power). Binding data were analysed using MO. Affinity Analysis software, where the K_D_ affinity model was used with the On-Time evaluation set to 5 s. Initial binding checks were first conducted, and full binding curves were recorded for reliable K_D_ determination. Measurements were performed in triplicate [23].

#### 2.2.8. Molecular Docking

The M3 structure was modelled from the melittin crystal structure template (PDB ID: 2MLT; X-ray diffraction, 2.00 Å). Blind docking was performed using AutoDock Vina v1.2.7 implemented through UCSF Chimera version 1.18 to explore potential binding sites of doxorubicin on M3 without predefined grid constraints [24]. A grid box sufficient to encompass the entire peptide structure was defined, ensuring unbiased sampling of all possible binding regions. The top-ranked docking pose based on binding score was selected for further analysis. Non-covalent interactions between M3 and doxorubicin were characterized using BIOVIA visualizer [25]. The selected docking complex was subsequently subjected to molecular dynamics simulations in explicit aqueous solvent.

#### 2.2.9. MD Simulations

MD simulations were performed using the GROMACS 2021.5 package [10]. The Amber *ff*99sb-ILDN [26] force field was applied for the peptide and protein molecules, and the TIP3P [27] water model was used to solvate the system. Topology files for the ligand (doxorubicin) were generated using ACPYPE [28] to ensure compatibility with the Amber force field. The top-ranked melittin analogue-doxorubicin complex obtained from docking studies was prepared for MD simulations by centering the complex in a cubic simulation box with periodic boundary conditions, ensuring a minimum distance of 1.0 nm between the solute and the box edges. The system was solvated with TIP3P water molecules, and Na^+^ and Cl^−^ ions were added to neutralize the system and simulate physiological ionic strength. The system was energy minimized using the steepest descent algorithm to eliminate steric clashes and optimize geometry. Equilibration involved heating to 300 K in the NVT ensemble over 300 ps, followed by 50 ps of equilibration at 300 K and 1 bar in the NPT ensemble. Following this initial equilibration, a 200 ns production MD simulation was performed at 300 K and 1 bar. Simulations used a time step of 2 fs. Long-range electrostatics were treated using the Particle Mesh Ewald (PME) method [29], and bond lengths involving hydrogens were constrained using the LINCS algorithm [30]. Post-simulation analyses were performed using GROMACS functionality. Root mean square deviation (RMSD) was calculated using the backbone atoms (C, CA, and N) of the peptide relative to the initial structure to assess structural stability. Radius of gyration (Rg) was computed to evaluate overall compactness. Root mean square fluctuation (RMSF) was calculated per residue based on Cα atoms to assess residue-level flexibility. α-helical content was determined using DSSP [31]. The center of mass (COM) distance between tryptophan residues and doxorubicin was calculated to monitor binding proximity over time. Structural conformations were visualized using ChimeraX [32], and dynamic parameters were plotted using GraphPad Prism. Non-covalent interactions between each melittin analogue and doxorubicin were further characterized using BIOVIA Discovery Studio Visualizer [25].

#### 2.2.10. Binding Free Energy Calculation Using MM-PBSA Method

The binding free energies of doxorubicin with melittin analogues were calculated using the external tool *g_mmpbsa*, which is based on the Molecular Mechanics Poisson–Boltzmann Surface Area (MM-PBSA) methodology [33]. This method estimates the total binding energy from endpoint calculations, computing various contributions, including van der Waals, electrostatic, polar solvation, and non-polar (apolar) solvation terms. Per-residue energy decomposition analysis was conducted using an in-house Python 3.14.6 script to identify key residues contributing to ligand binding within the interaction interface. The entire equilibrated 100 ns MD trajectory was used for the MM-PBSA calculations, which were performed using the following equation:

∆G_bind_ = G_complex_ − (G_peptide_ + G_ligand_) (3)

where G_complex_ represents the total free energy of the binding complex, while G_peptide_ and G_ligand_ denote the total free energies of the peptide and doxorubicin, respectively.

#### 2.2.11. Cell Viability

Cell viability was evaluated using the CellTiter-Blue assay, which measures the reduction of resazurin to the fluorescent product resorufin by metabolically active cells [34]. Cells were seeded in 96-well plates at a density of 10,000 cells per well in 100 µL of culture medium and allowed to attach for 24 h. Wells in the first column were used as untreated controls. Treatment solutions were prepared by diluting peptide and doxorubicin stock solutions (100 µM) in culture medium to obtain the desired concentrations. After removal of the culture medium, 100 µL of the treatment solutions were added to each well, and the cells were incubated for a further 24 h. Following treatment, 20 µL of CellTiter-Blue reagent was added to each well, and the plates were incubated for 3 h. Resorufin fluorescence was measured using a Clariostar Plus plate reader (BMG LABTECH, Ortenberg, Germany) with excitation at 545 nm and emission at 600 nm (bandwidth 20 nm and 40 nm, respectively). Cell viability (%) was calculated using the following equation:

% Cell viability = (F_t_ − F_b_)/(F_c_ − F_b_) × 100 (4)

where F_t_ is the fluorescence intensity of treated cells, F_c_ is the fluorescence intensity of untreated cells, and F_b_ is the fluorescence intensity of blank wells containing culture medium and CellTiter-Blue reagent without cells. Fluorescence values were corrected by subtracting the background signal obtained from blank wells.

#### 2.2.12. Immunofluorescence

H69AR cells were seeded at 30,000 cells/well in Ibidi 8-well chamber slides (300 µL/well) and incubated overnight at 37 °C, 5% CO_2_. Cells were fixed with 3.7% formalin for 5 min, washed with PBS, permeabilized with 0.1% Triton X-100 for 2 min, and blocked with 1% BSA/PBS for 1 h at room temperature. Primary antibodies against Pgp and MRP1 (1:400 in 1% BSA/PBS) were applied for 2 h at room temperature, followed by three PBS washes. Alexa Fluor 488-conjugated anti-mouse secondary antibody (1:500) was added for 1 h in the dark, and cells were washed again. Nuclei were stained with DAPI (1 µg/mL, 3 min). Slides were mounted with antifade mounting medium, sealed with coverslips, and imaged using a Cell Discoverer 7 automated confocal microscope (Carl Zeiss Microscopy GmbH, Jena, Germany).

#### 2.2.13. Calcein AM Assay

The calcein acetoxymethyl ester (calcein-AM) assay was used to assess Pgp and MRP1 efflux activity [35]. Calcein-AM is a non-fluorescent, membrane-permeable substrate that is hydrolysed intracellularly to fluorescent calcein, while active efflux pumps reduce intracellular fluorescence. Cells were seeded at 25,000 cells per well in 96-well plates and incubated overnight. The next day, the medium was replaced with FBS-free medium and cells were incubated for 30 min. Verapamil (25 µM) was added 30 min prior to treatment as a positive control. Peptide and calcein-AM were prepared at a 1:1 ratio (final concentration 1 µM each) and pre-incubated for 1 h in FBS-free medium before addition to the viable cells. Cells were then exposed to the peptide–calcein AM mixture for 1 h. Wells without peptide served as negative controls. After incubation, the solution was removed, cells were washed twice with cold PBS, and 200 µL cold PBS was added. Fluorescence was measured using a Clariostar Plus plate reader (BMG LABTECH, Ortenberg, Germany) (excitation 483 nm, emission 520 nm). Calcein retention (%) was calculated using the following formula:

(5) $$\mathrm{Calcein}\;\mathrm{retention}\;\%\;=\frac{\mathrm{Fluorescence}\;\mathrm{intensity}\;\mathrm{of}\;\mathrm{treated}\;\mathrm{cells}}{\mathrm{Fluorescence}\;\mathrm{intensity}\;\mathrm{of}\;\mathrm{untreated}\;\mathrm{cells}}\;\times \;100$$

#### 2.2.14. Doxorubicin Accumulation

Doxorubicin accumulation was assessed in H69AR cells using time-lapse imaging. Cells were seeded in ibidi µ-Slide 8-well chambers at 30,000 cells per well and allowed to adhere for 24 h. Nuclei were stained with Hoechst (2.5 µM, 10 min), after which the dye-containing medium was replaced with fresh culture medium. Cells were then treated with either doxorubicin alone (5 µM) or a combination of doxorubicin (5 µM) and M3 peptide (0.5 µM). Doxorubicin intracellular accumulation in the presence and absence of M3 was monitored using a Cell Discoverer 7 automated confocal microscope (Carl Zeiss Microscopy GmbH, Jena, Germany) in time-lapse mode, with images acquired every 30 min for 3 h. Live-cell confocal images were quantified using ImageJ version 1.54g. Images were acquired using consistent microscope settings across all treatment groups and time points. Signal saturation was avoided during image acquisition. For each field of view, square regions of interest (ROIs) of identical size were placed over intact cell-associated areas to quantify fluorescence intensity, while avoiding cell-free background regions. The same ROI size and selection criteria were applied consistently across all samples, including doxorubicin-only and M3–doxorubicin treatment groups. Background fluorescence was measured from a cell-free region within the same image and subtracted from the raw fluorescence intensity. Corrected mean fluorescence intensity was then calculated for each field of view and averaged across three fields per well from three independent experiments. Statistical comparison between groups was performed using an unpaired t-test with Welch’s correction.

#### 2.2.15. Bliss Independent Synergy Analysis

Synergistic interactions between M3 and doxorubicin were evaluated using the Bliss independence model, which assumes that two agents act through independent mechanisms. The expected additive effect for a given combination was calculated as:

E_Bliss_ =E_A_ + E_B_ − (E_A_ × E_B_) (6)

where E_A_ and E_B_ represent the fractional inhibitory effects (0–1) of each agent alone. The Bliss ratio was determined as:

(7) $$\mathrm{Bliss}\;\mathrm{ratio}\;=\;\frac{E_{\mathrm{Bliss}}}{E_{\mathrm{observed}}}$$

Interpretations are Bliss ratio < 1: synergy, Bliss ratio = 1: additive effect, and Bliss Ratio > 1: antagonism. This model is appropriate because M3 and doxorubicin operate through mechanistically independent pathways [36].

#### 2.2.16. Statistical Analysis

Statistical analysis was performed using GraphPad Prism 10 (GraphPad Software, San Diego, CA, USA). Half-maximal effective concentration (EC_50_) and half-maximal inhibitory concentration (IC_50_) values were determined by non-linear regression using a variable-slope four-parameter logistic model. For EC_50_ determination, membrane leakage data were fitted using the “[Agonist] vs. normalized response—Variable slope” model. For IC_50_ determination, cell viability data were fitted using the “[Inhibitor] vs. normalized response—Variable slope” model. Responses were normalized prior to curve fitting, and the Hill slope was fitted as a variable parameter. IC_50_ and EC_50_ values were reported as mean ± SD from independent experiments. Differences between two independent groups were analysed using an unpaired *t*-test with Welch’s correction, and comparisons among three or more groups were assessed using one-way ANOVA followed by Tukey’s post hoc test. Data are presented as mean ± SD, with *p* < 0.05 considered statistically significant.

## 3. Results and Discussion

### 3.1. Rationale for Design of Hybrid Peptide

Peptide M3 was designed to contain the drug-binding motif of Popkov’s A4 peptide [9] while retaining key melittin properties (Table 1). Four residues within positions 11–15 were substituted, with threonine at position 11 replaced by leucine, glycine at position 12 by tryptophan, leucine at position 13 by serine, and alanine at position 15 by tryptophan, generating a WSPW motif spanning residues 12–15. As a result, residues 11–19 in M3 share seven identical residues with the Popkov A4 sequence (LWSPWXXSW), enabling integration of a putative drug-binding motif within the melittin framework. The conserved Pro14 residue was retained to preserve the characteristic helix kink, which separates the N- and C-terminal regions and contributes to conformational flexibility during membrane interaction [37]. Peptide length and net charge were maintained to minimize disruption to membrane-binding properties.

### 3.2. Molecular Docking of M3 with Doxorubicin

**Figure 1:**
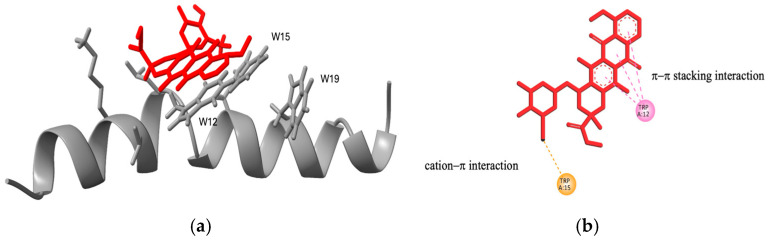
Docking and interaction analysis of M3–doxorubicin complex. (**a**) Top-ranked docking pose of doxorubicin (red) with melittin analogue (grey) based on docking score. (**b**) 2D visualization of non-covalent interactions generated by Biovia visualizer software.

Blind docking of M3 with doxorubicin predicted from the highest-scoring poses suggested that both tryptophans in the WXXW motif contribute to binding. W12 formed π–π stacking with the anthraquinone ring, while W15 engaged in a cation–π interaction with the protonated daunosamine group (Figure 1), supporting the WXXW motif as a drug-binding region.

### 3.3. Physical Properties

M3 is a 26-residue, strongly basic peptide (pI 12.43; net charge +6 at physiological pH), retaining the length and overall physicochemical properties of native melittin. Incorporation of the WXXW motif by substituting TGLPA with LWSPW increases the molecular weight from 2846.45 to 3076.95 Da. M3 shows higher mean helical hydrophobicity (H = 0.66) than melittin (H = 0.51), but a slightly lower hydrophobic moment (μH = 0.352 vs. 0.394), reflecting a more even distribution of hydrophobic residues rather than a defined amphipathic face (Figure S1). This may modestly reduce amphipathicity and membrane insertion propensity. The helical wheel further indicates clustering of the three tryptophan residues.

### 3.4. Peptide Synthesis and Characterization

**Figure 2:**
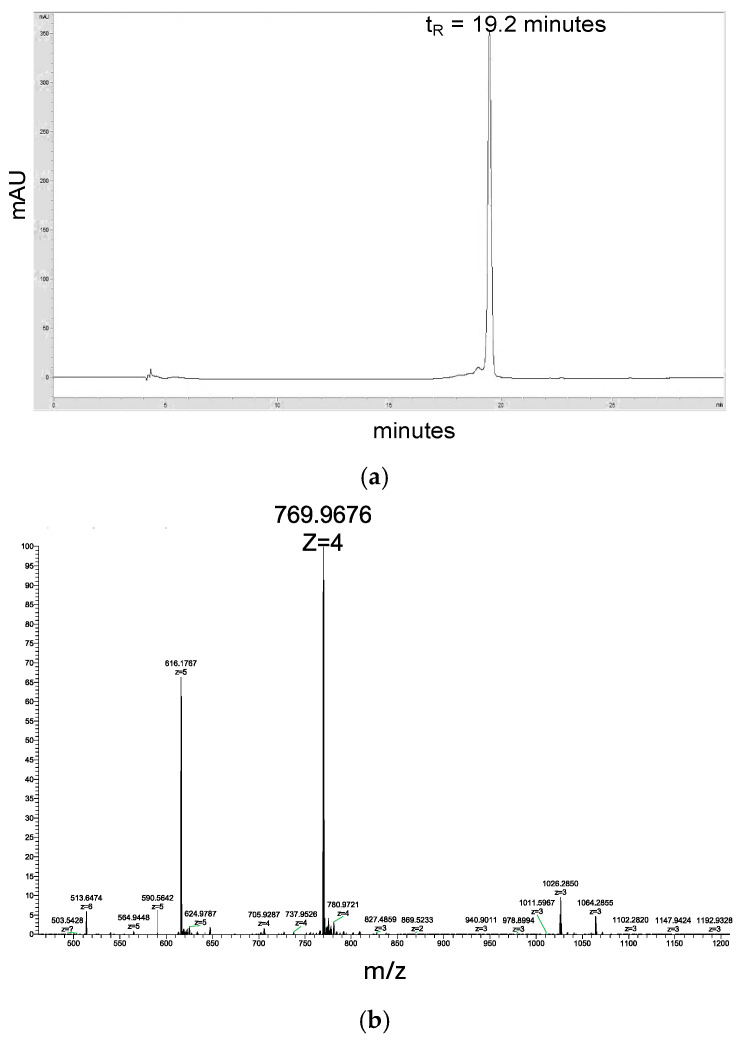
Characterization of M3 peptide synthesized via SPPS. (**a**) R-HPLC chromatogram of the purified peptide showing single peak with retention time of 19.2 min. (**b**) ESI-MS spectrum of M3 showing the dominant ion at *m*/*z* 769.97 (z = 4).

M3 was synthesized using Boc solid-phase peptide synthesis and purified by reverse-phase HPLC. The purified peptide eluted as a single major peak at 19.2 min, confirming high purity (Figure 2a). Electrospray mass spectrometry verified the peptide identity, showing a dominant ion at *m*/*z* 769.97 (z = 4), consistent with the expected molecular weight of 3076.95 Da (Figure 2b).

### 3.5. Functional Analysis of M3

M3 and melittin showed almost identical liposome lysis profiles (EC_50_: 12.58 ± 1.25 vs. 9.81 ± 0.48 nM; Figure S2A), with a modest but statistically significant reduction for M3, consistent with its lower amphipathicity. CD spectra revealed similar structural transitions from random coil in water (195–200 nm) to α-helix in 50% TFE (208 and 222 nm; Figure S2B). Helical content increased to 85.9% (M3) and 89.7% (melittin), indicating that M3 adopts a membrane-active helical conformation. This structural transition supports its ability to associate with and penetrate lipid bilayers.

### 3.6. Biophysical Characterizations of M3-Doxorubicin Binding

**Figure 3:**
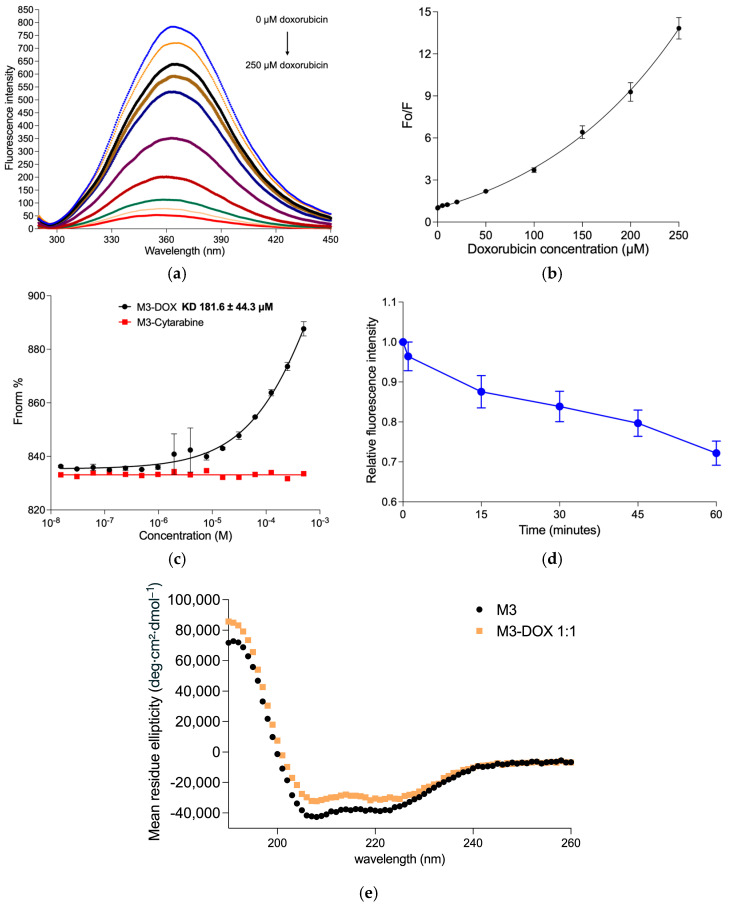
Biophysical characterization of M3-doxorubicin binding. (**a**) Tryptophan fluorescence emission spectra of M3 recorded in the presence of increasing concentrations of doxorubicin. The spectra are arranged from the lowest doxorubicin concentration (0 µM; uppermost curve) to the highest doxorubicin concentration (lowermost curve), as indicated in the inset. (**b**) Stern–Volmer plot of M3-doxorubicin. (**c**) MST binding curves for Cy5-labeled M3 with doxorubicin, with cytarabine included as a non-binding control. (**d**) Time-dependent tryptophan fluorescence measurements of 1 µM M3 following the addition of 1 µM doxorubicin at a 1:1 ratio over 60 min (**e**) CD spectra of M3 in 50% TFE in the absence and presence of 50 µM equimolar doxorubicin.

The binding interaction of M3 with doxorubicin was investigated using tryptophan quenching and microscale thermophoresis (MST) (Figure 3). Increasing doxorubicin concentration resulted in progressive quenching of tryptophan fluorescence (Figure 3a), with an upward-curving Stern–Volmer plot suggesting combined dynamic and static quenching mechanisms (Figure 3b) [38]. Analysis using the modified Stern–Volmer model yielded a dissociation constant (K_D_) of 238.9 ± 38.2 µM. MST with Cy5-labeled M3 provided a comparable K_D_ of 181.6 ± 44.3 µM, while cytarabine showed no binding, confirming specificity (Figure 3c). These results indicate consistent mid-micromolar affinity, characteristic of moderate and reversible non-covalent binding.

Time-dependent quenching further supported the occurrence of M3–doxorubicin interaction beyond an immediate quenching event. M3 showed a rapid initial decrease in fluorescence, followed by a slower stabilization phase over approximately 45 min (Figure 3d), with significant time-dependent quenching (*p* < 0.001). This pattern is consistent with initial peptide–drug contact followed by gradual structural rearrangement or stabilization of the M3–doxorubicin complex. CD spectroscopy further showed that M3 adopted a predominantly α-helical structure in 50% TFE, with an estimated helicity of 85.9% (Figure 3e). Upon addition of equimolar doxorubicin, helicity decreased to 59.1%, indicating partial structural perturbation without complete loss of secondary structure. Together, these findings suggest that doxorubicin binding promotes conformational adjustment of M3 while maintaining substantial secondary structure.

### 3.7. MD Simulation of the M3-Doxorubicin Complex

**Figure 4:**
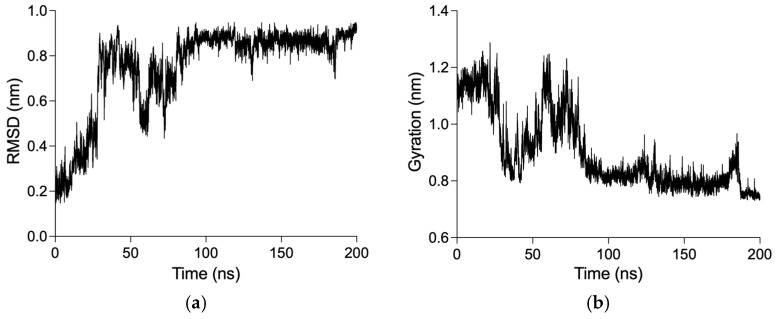

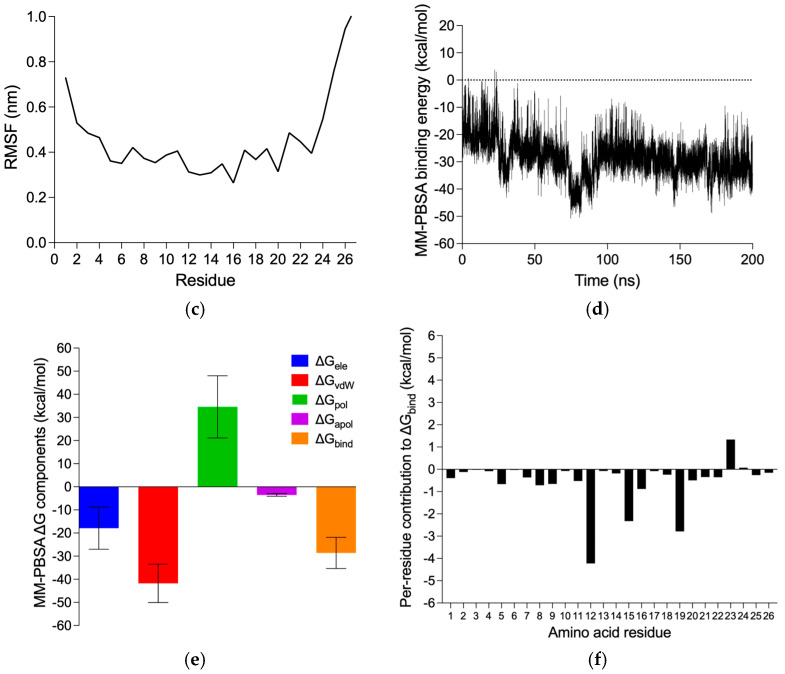
Molecular dynamics analysis of the M3–doxorubicin complex. (**a**) RMSD profile of the complex over the 200 ns simulation. (**b**) Radius of gyration plotted across the trajectory. (**c**) RMSF of M3 residues. (**d**) MM/PBSA binding free energy in (kcal/mol) over 200 ns production trajectory. (**e**) MM/PBSA binding energy component decomposition. (**f**) Per-residue contribution to the MM/PBSA binding free energy.

A 200 ns molecular dynamics (MD) simulation was performed to evaluate the stability and binding dynamics of the M3–doxorubicin complex and the role of tryptophan residues within the WXXW motif. Doxorubicin remained associated with M3 throughout the simulation. RMSD analysis indicated an initial rise within 30 ns, reflecting rapid structural adaptation, followed by moderate fluctuations between 50–100 ns and a subsequent plateau, indicating convergence to a stable conformation (Figure 4a). Consistent with this, the radius of gyration stabilized at ~0.8 nm after 100 ns, suggesting formation of a compact complex (Figure 4b). RMSF analysis showed higher flexibility at the N- and C-termini, while the central region remained more rigid (Figure 4c), consistent with reported melittin behaviour [39]. MM-PBSA analysis showed increasingly favourable binding over time, with stabilization after ~50 ns and more consistent negative values between 100–200 ns (Figure 4d). The average binding free energy was −28.6 ± 6.8 kcal mol^−1^, predicting a thermodynamically favourable interaction. Van der Waals interactions (−41.8 ± 8.3 kcal mol^−1^) were the dominant stabilizing force, supported by electrostatic contributions (−17.9 ± 9.1 kcal mol^−1^), while polar solvation opposed binding. Per-residue decomposition highlighted the key role of tryptophan residues, with W12 contributing most strongly (−4.2 kcal mol^−1^), followed by W19 (−2.8 kcal mol^−1^) and W15 (−2.3 kcal mol^−1^), indicating that aromatic interactions within the WXXW motif are central to stabilizing the complex.

#### 3.7.1. Progression of Binding from Surface Contact to More Enveloped State

**Figure 5:**
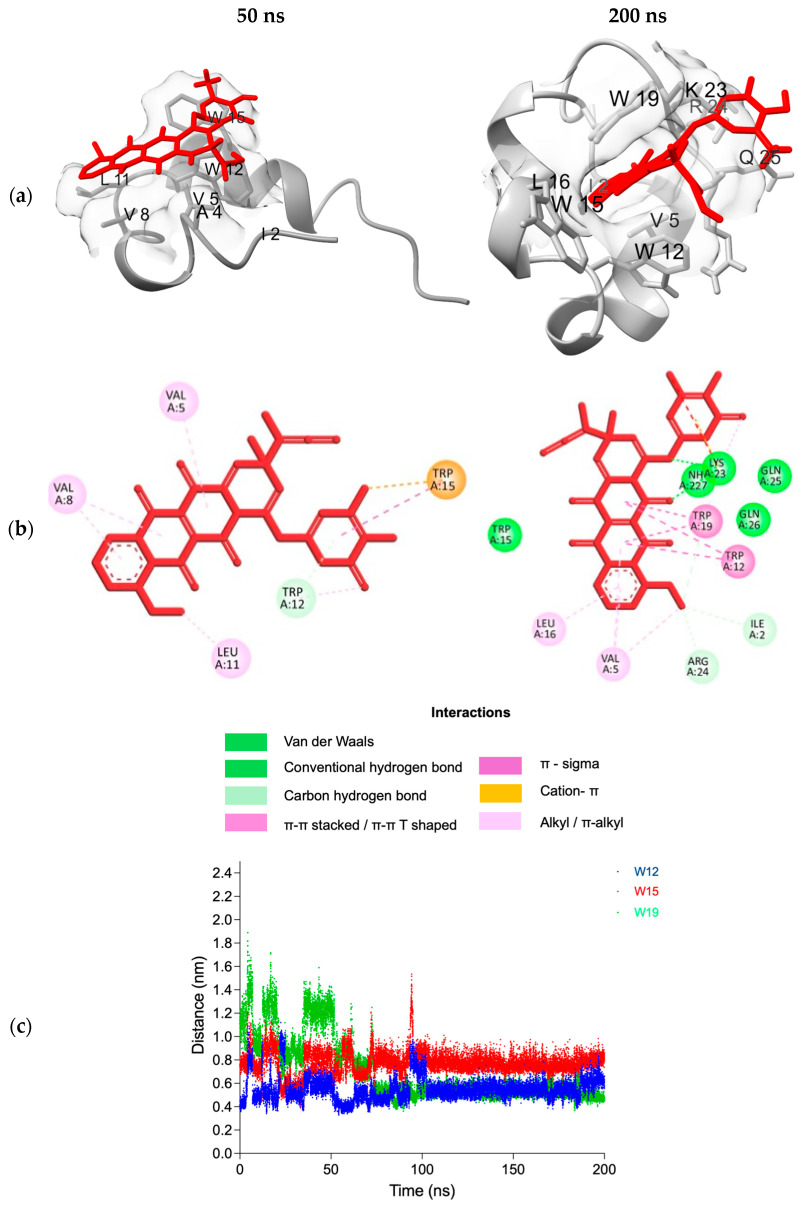
Interaction analysis of the M3–doxorubicin complex across the MD trajectory. (**a**) Cartoon and surface representations of the M3–doxorubicin interaction at 50 ns and 200 ns, highlighting key interacting residues. Doxorubicin (red) with M3 peptide (grey). (**b**) Two-dimensional interaction maps generated using BIOVIA Discovery Studio, showing residue–ligand contacts at 50 ns (left) and 200 ns (right) of the MD simulation. Non-covalent interactions are color-coded as follows: green (conventional hydrogen bond); pale green (carbon hydrogen bond); purple (π-sigma); light pink (alkyl and π-alkyl interactions); dark pink (π-π stacked and π-π T-shaped interactions); orange (π-cation). Residues involved only in van der Waals contacts are shown in green fill without a connecting line (**c**) Center of mass distances between doxorubicin and the three tryptophan residues (W12 (blue), W15 (red) and W19 (green)) over 200 ns.

The molecular dynamics simulation revealed a dynamic progression in which doxorubicin initially interacted with exposed surface regions of M3. Over time, the peptide reorganized its conformation to wrap around the drug, stabilizing key interactions (Figure S3). W12 anchored the doxorubicin through a T-shaped π–π interaction, aided by a cation-π contact from W15 (Figure 5b). As the simulation progressed, W19 approached and engaged doxorubicin alongside W12. The interaction further deepened as K23 formed a stable hydrogen bond with doxorubicin, while W12 and W19 maintained π–π stacking and W15 provided peripheral support. By 200 ns, K23, W12, and W19 remained the main interacting residues, supported by additional contacts from both termini (Figure 5b). The distances between doxorubicin and the tryptophan residues further support this progressive stabilization (Figure 5c). W19, which initially resided more than 1 nm away from the drug, moved progressively closer between 50 and 100 ns, eventually converging with W12 within the 0.4–0.6 nm range as the peptide adjusted its conformation. The consistent proximity of W12 and W19 at these distances indicates persistent π–π stacking on the doxorubicin anthracycline ring, while W15 remained slightly more distant (0.6–0.8 nm) but engaged in weaker secondary contacts such as π-donor hydrogen-bond-like and van der Waals interactions. This binding progression aligns with time-dependent quenching, involving an initial encounter complex that subsequently undergoes conformational stabilization.

#### 3.7.2. Mechanistic Interpretation of Computational Analysis Regarding Potential of M3 to Evade MDR Efflux Proteins

These computational findings imply that in the bound state, peptide residues interact with the anthracycline and daunosamine moieties of doxorubicin, which correspond to regions implicated in Pgp and MRP1 recognition [40,41,42,43]. The evolution of these contacts indicates that M3 engages similar chemical features associated with efflux recognition, suggesting a potential masking effect. By interacting with these functional groups, the peptide may partially limit their accessibility to transporters. This enveloping behavior therefore reflects not only structural adaptation for stability but also a possible transient shielding mechanism that could reduce efflux susceptibility and support intracellular retention. Reported MM-PBSA and MM-GBSA binding free energies for doxorubicin with Pgp and MRP1 range from approximately −11 to −33 kcal mol^−1^ in membrane systems [44,45,46], while the M3–doxorubicin complex in bulk water shows a comparable value (−29 kcal mol^−1^). Although the environments differ, the similar magnitudes in computed affinity support the idea that M3 can stabilize doxorubicin sufficiently to partially mask key interaction sites. This does not imply competitive binding with transporters but suggests a transient shielding effect that may reduce immediate efflux recognition.

### 3.8. Calcein AM Assay Suggests Reduced MRP-Mediated Efflux in H69AR Cell Line

**Figure 6:**
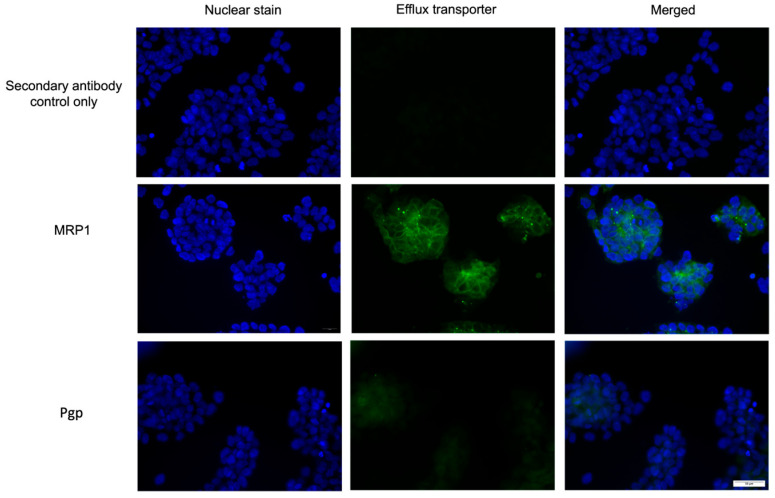
Immunofluorescence staining of MRP1 and Pgp in the H69AR cell line. Cells were stained with primary antibodies against MRP1 and Pgp (1:400) followed by Alexa Fluor 488-conjugated anti-mouse secondary antibody (1:500). Nuclei were counterstained with DAPI (blue). Strong membrane-localized fluorescence was observed for MRP1 (green), whereas Pgp (green) showed weak staining, indicating low expression in H69AR cells. Images were acquired using confocal microscopy. Scale bar = 50 µm.

H69AR, a human small-cell lung carcinoma cell line, was selected as the primary model due to its well-defined doxorubicin-resistant phenotype. In viability assays, H69AR exhibited a significantly higher IC_50_ for doxorubicin (21.1 ± 5.6 µM) compared to the parental H69 line (2.87 ± 0.51 µM) (Figure S4A), corresponding to a resistance index (RI) of 7.4 (Figure S4B). H69AR was therefore prioritized for subsequent mechanistic and uptake studies. Immunofluorescence staining further confirmed overexpression of the ABC transporter MRP1, with strong membrane-localized fluorescence (Figure 6), while Pgp staining was weak. These findings indicate that MRP1 is the predominant efflux transporter in H69AR cells. Transporter function was subsequently assessed using the calcein-AM assay.

Calcein-AM serves as a validated functional probe for multidrug resistance, as it is a substrate for Pgp and MRP1 [35,47]. This assay employs calcein-AM, a hydrophobic and membrane-permeant non-fluorescent esterase substrate, which can enter cells and undergo intracellular esterase-mediated hydrolysis to generate fluorescent calcein. Therefore, increased intracellular fluorescence reflects enhanced net intracellular calcein accumulation, which may arise from reduced transporter-mediated efflux of calcein-AM. Given the predominant expression of MRP1 in H69AR cells, the assay primarily reflects MRP1-mediated efflux. At the sub-lytic peptide concentration used and with only one hour of treatment time, cell viability is maintained, and membrane integrity is less likely to be significantly compromised.

**Figure 7:**
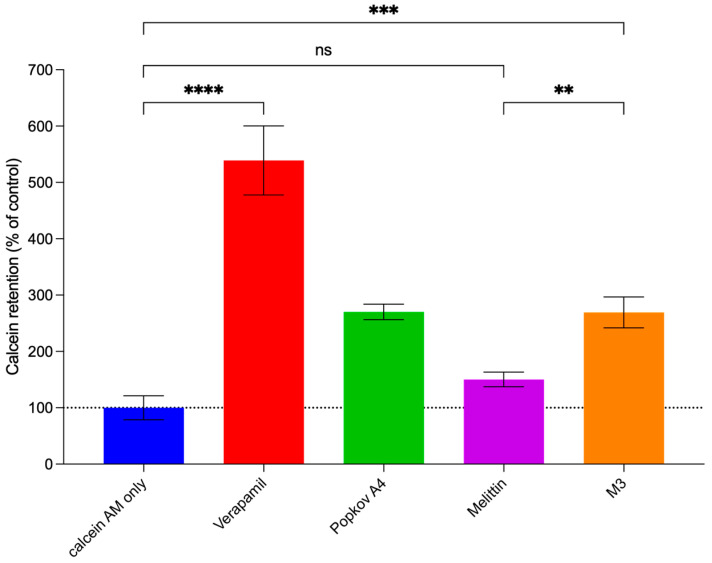
Calcein AM assay in H69AR cells following treatment with melittin analogues. Calcein-AM (1 µM), a substrate of Pgp and multidrug resistance-associated protein 1 (MRP1), was used as a functional probe of efflux activity. After entering the cells, Calcein-AM is hydrolysed by intracellular esterases to fluorescent calcein, which is retained in the cytoplasm unless exported by efflux transporters. Cells were incubated with Calcein-AM (1 µM) and treated for 1 h with peptides (1 µM) or verapamil (25 µM). Following treatment, cells were washed twice with PBS to remove extracellular dye, PBS was added, and intracellular fluorescence was measured. As H69AR cells predominantly overexpress MRP1, the assay readout primarily reflects MRP1-mediated efflux. The x-axis represents calcein retention (%), corresponding to intracellular fluorescence from calcein generated after Calcein-AM conversion. Data are presented as mean ± SD from three independent experiments, with statistical analysis performed using one-way ANOVA followed by Tukey’s post hoc test (** *p* < 0.01; *** *p* < 0.001; **** *p* < 0.0001; ns, not significant).

Cells were treated under sub-lytic conditions with a 1:1 molar ratio of peptide to calcein-AM. As shown in Figure 7, both M3 and the Popkov A4 peptide significantly increased intracellular calcein retention after 1 h, producing approximately a two- to three-fold increase compared to control (*p* < 0.001, Tukey’s test). Verapamil produced the greatest effect (~four- to five-fold increase; *p* < 0.0001), while native melittin showed a modest, non-significant change (*p* = 0.38). No significant difference was observed between M3 and A4 (*p* > 0.99), although both showed higher retention than melittin (*p* = 0.008 and *p* = 0.0078, respectively). Since native melittin possesses membrane-active properties but lacks the WXXW drug-binding motif, its comparatively weaker effect than M3 indicates that membrane perturbation alone does not fully account for the increased calcein retention. The enhanced activity of both M3 and the Popkov A4 (membrane-inactive) peptides suggests that the WXXW motif may provide an additional substrate-interaction mechanism that contributes to reduced efflux-associated loss of calcein-AM. Verapamil remained significantly more effective than all peptide groups (*p* < 0.0001). These results indicate that M3 enhances intracellular calcein retention in an MRP1-overexpressing model, with an effect greater than melittin but lower than positive control verapamil.

Together, these findings suggest that M3 enhances intracellular calcein retention through partial efflux modulation rather than classical transporter inhibition alone. This effect may arise from a combination of membrane-associated activity and transient substrate association. As a melittin-derived peptide, M3 may influence calcein-AM uptake or the local membrane environment, while the incorporated WXXW motif may promote transient interactions with hydrophobic small-molecule substrates, potentially reducing the pool of freely available substrate for MRP1 recognition. This interpretation is indirectly supported by molecular dynamics simulations showing sustained association of doxorubicin with the WXXW-containing region of M3. Although the simulation was performed with doxorubicin rather than calcein-AM, it provides supporting evidence for the capacity of this motif to associate with hydrophobic small molecules, which may be relevant to calcein-AM as a transporter substrate.

### 3.9. Differential Cytotoxic Response of H69AR and Normal Lung Epithelial BEAS-2B Cells to M3

The cytotoxic response to M3 was compared between H69AR cancer cells and BEAS-2B normal epithelial cells to evaluate whether M3 exhibited differential toxicity toward cancer and normal cells within the same concentration range. M3 demonstrated potent, dose-dependent cytotoxicity in H69AR cells, with an IC_50_ of 1.58 ± 0.10 µM. In contrast, normal BEAS-2B lung epithelial cells maintained over 80% viability at 2 µM (Figure S5). This concentration exceeded the H69AR IC_50_ by approximately 1.3-fold, indicating a differential acute cytotoxic response between H69AR and BEAS-2B cells under the tested conditions. As BEAS-2B viability did not fall below 50% within the tested concentration range, a BEAS-2B IC_50_ value could not be determined, and a formal IC_50_-based selectivity index could not be precisely calculated. Nevertheless, the preservation of over 80% BEAS-2B viability at 2 µM supports an apparent selectivity window within the tested concentration range.

This differential cytotoxic response may be explained by differences in the membrane properties of malignant and normal cells. As M3 is derived from melittin and retains cationic amphipathic membrane-active characteristics, its interaction with cells is likely influenced by membrane charge, fluidity, and lipid organization. By analogy with melittin, the positive charge of M3 is expected to facilitate electrostatic interactions with negatively charged lipid headgroups, promoting membrane binding and insertion. Cancer cell membranes have been reported to exhibit increased surface negative charge, partly due to externalization of phosphatidylserine and other anionic lipids [48,49], which can enhance electrostatic attraction and promote melittin binding [50,51,52]. Cancer cell membranes are also often described as more fluid and less tightly packed, conditions that may facilitate peptide adsorption, insertion, and membrane permeabilization [53,54,55]. In addition, structural features of malignant cell membranes, including increased curvature and surface irregularity, may reduce local mechanical resistance and lower the energetic barrier for melittin insertion [56,57,58,59,60,61]. Therefore, the stronger cytotoxic response observed in H69AR cells compared with BEAS-2B cells may reflect a greater permissiveness of cancer cell membranes to M3 interaction. Further studies assessing membrane composition, membrane integrity, and longer-term cytotoxicity in additional normal-cell models would help to more precisely define the selectivity and safety profile of M3.

### 3.10. M3 Enhanced Doxorubicin Accumulation in Multidrug Resistant H69AR Cells

**Figure 8:**
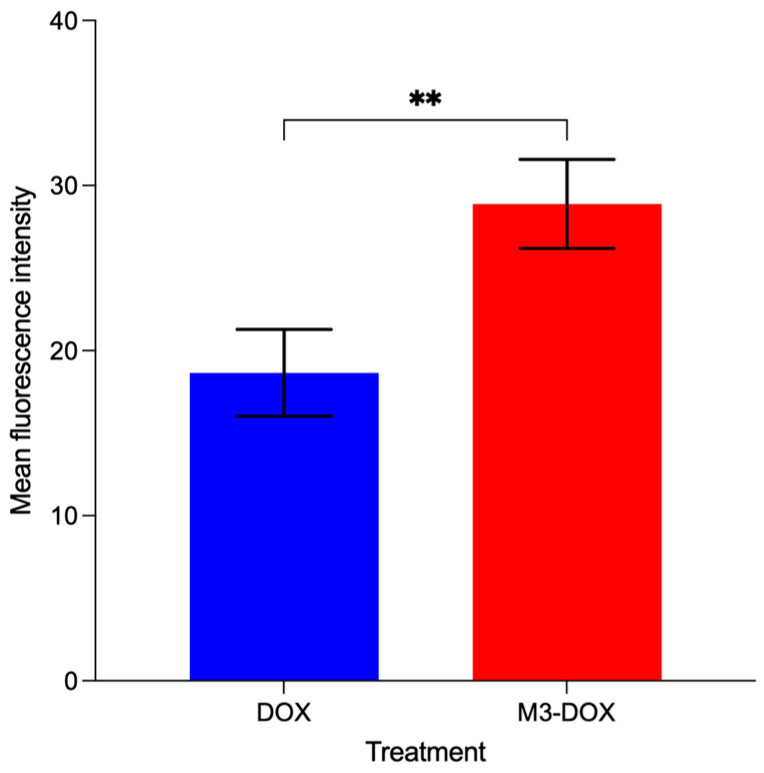
Whole-cell mean fluorescence intensity (MFI) of intracellular doxorubicin at the 3-h endpoint (mean ± SD, n = 3; Welch-corrected unpaired *t*-test, *p* = 0.0093, ** *p* < 0.01).

To further investigate the effect of M3 peptide on drug transport in an MDR cell line, doxorubicin accumulation in the H69AR cell line was monitored using live-cell confocal imaging under non-lytic conditions. Concentrations of 0.5 µM M3 and 5 µM doxorubicin were selected to minimize peptide-induced cytotoxicity, as 0.5 µM M3 maintained approximately 80% cell viability after 24 h. This was considered suitable for the 3 h imaging assay to assess doxorubicin retention while minimizing peptide-induced membrane damage. The 5 µM doxorubicin dose also provided the optimal fluorescence intensity for reliable confocal detection and quantification. Time-lapse confocal microscopy over 3 h (30-min intervals) revealed a time-dependent increase in intracellular doxorubicin in both groups (Figure S6A). In the doxorubicin-only condition, MFI rose from 1.23 ± 0.31 at 30 min to 18.66 ± 2.62 at 180 min. In the presence of M3, uptake increased more sharply, from 1.13 ± 0.46 to 28.88 ± 2.30 over the same period, with differences becoming apparent after 150 min (Figure S6B). At the 3-h endpoint, M3 further enhanced doxorubicin accumulation (*p* = 0.0093; Figure 8). The mean difference between groups was 10.23 MFI units, with a 95% confidence interval of 4.19 to 16.26, supporting a significant increase in doxorubicin-associated fluorescence in the presence of M3. The mean fluorescence intensity of cells treated with M3–DOX was 1.6-fold higher than DOX alone, suggesting enhanced intracellular retention. The observed enhancement in intracellular doxorubicin accumulation cannot be taken to reflect active or carrier-mediated transport by M3. Instead, the data are more consistent with a transient and reversible M3–doxorubicin association altering cellular or uptake dynamics to promote drug accumulation and retain it within cells.

**Figure 9:**
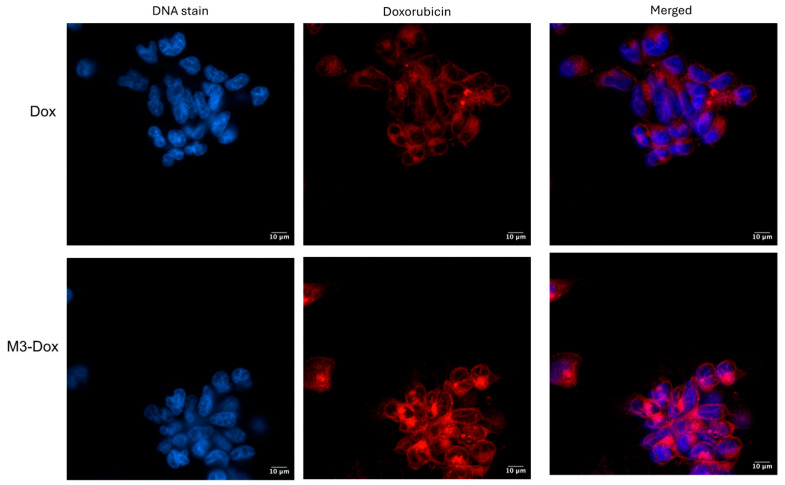
Confocal images showing the intracellular distribution of 5 µM doxorubicin (red) after 3 h in the absence and presence of 0.5 µM M3, with nuclear staining (blue) included to visualize localization. Scale bar =10 µM.

Endpoint confocal images with nuclear staining (Figure 9) showed peripheral membrane-associated doxorubicin in both groups, with red–blue overlap exhibiting pink colour indicating nuclear localization. Qualitatively, cells treated with the M3–doxorubicin combination exhibited a visibly brighter intracellular (cytoplasmic) signal than cells treated with doxorubicin alone.

### 3.11. M3-Doxorubicin Combination Shows Bliss-Indicated Synergistic Cytotoxicity in H69AR Cell

**Figure 10:**
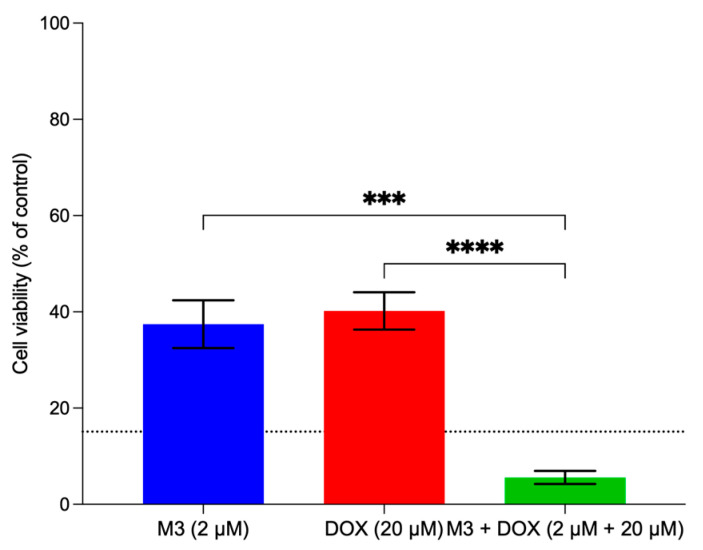
Combination treatment of 2 µM peptide and 20 µM doxorubicin assessed for synergistic effects after 48 h treatment. Statistical analysis was performed using one-way ANOVA followed by Tukey’s multiple comparison test. A dotted horizontal line represents the Bliss-expected additive effect. Values below the line indicate synergy, whereas values above the line indicate antagonism. Statistical significance is indicated as *** *p* = 0.0001; **** *p* < 0.0001.

Selected fixed-dose M3–doxorubicin combinations were evaluated in multidrug-resistant H69AR cells at 24 and 48 h using the CellTiter-Blue assay and Bliss independence analysis. Bliss analysis was used because M3 and doxorubicin are proposed to act through distinct but complementary mechanisms [36], with full interaction values provided in Supplementary Figure S7 and Table S1. The fixed-dose combination with the greatest Bliss-derived interaction value is shown in Figure 10.

At 48 h, 2 µM M3 and 20 µM doxorubicin reduced H69AR cell viability to 37.4 ± 4.5% and 40.2 ± 3.9%, respectively, with no significant difference between the two single-agent treatments (mean difference: −2.78 percentage points; 95% CI: −12.11 to 6.55; *p* = 0.6524). The combination treatment further reduced viability to 5.6 ± 1.3%, which was significantly lower than M3 alone (mean difference: 31.85 percentage points; 95% CI: 22.52 to 41.18; *p* = 0.0001) and doxorubicin alone (mean difference: 34.62 percentage points; 95% CI: 25.29 to 43.96; *p* < 0.0001). Bliss independence analysis showed a positive interaction value of 0.90 ± 0.02, indicating that this combination exceeded the expected additive effect. Nevertheless, because the analysis was based on selected fixed-dose conditions rather than a full dose–response matrix, this interaction is described as Bliss-indicated synergy under defined conditions rather than definitive pharmacological synergy.

Bliss-derived values generally decreased from 24 h to 48 h, suggesting that the interaction between M3 and doxorubicin became more evident with prolonged exposure. At 48 h, combinations containing 10–20 µM doxorubicin produced values below the additivity threshold at both tested M3 concentrations, consistent with Bliss-defined synergy under these conditions. The stronger Bliss-defined interaction observed at later time points may reflect the proposed complementary mechanisms of M3 and doxorubicin. M3 may contribute membrane-associated activity and increased intracellular doxorubicin accumulation, while doxorubicin induces delayed DNA-damage-mediated cytotoxicity [62].

However, the observed additive or synergistic cytotoxic effect between M3 and doxorubicin cannot be fully explained by reversible M3–doxorubicin complex formation alone. M3 and doxorubicin have distinct primary mechanisms, involving membrane perturbation and DNA damage, respectively, which may provide multiple points of cellular stress. M3 may enhance intracellular doxorubicin accumulation by increasing membrane permeability and partially reducing P-glycoprotein- or MRP1-mediated efflux, potentially through transient peptide–drug association or alteration of the membrane environment [37]. In addition, melittin-related peptides have been reported to induce downstream cytotoxic effects, including reactive oxygen species generation, mitochondrial dysfunction, and inhibition of pro-survival signalling pathways such as PI3K/Akt and NF-κB [63]. Therefore, the enhanced cytotoxicity observed in H69AR cells may reflect a combination of improved doxorubicin accumulation and complementary cellular stress mechanisms. While evasion of drug efflux appears to contribute to the observed enhancement in cytotoxicity, other mechanisms, including changes in membrane permeability, may also play a role. Further mechanistic studies, including membrane integrity assays such as LDH release and propidium iodide uptake, as well as transporter genetic knockdown models, are warranted to delineate the actual relative contributions of membrane perturbation and efflux inhibition.

### 3.12. Caspase 3/7 Response to M3-Doxorubicin Combination

Caspase-3/7 activity was measured after 48 h to assess whether the enhanced cytotoxicity observed with M3–doxorubicin combinations was associated with caspase-dependent apoptosis. Activity was expressed as fold-change relative to the untreated control. As shown in Supplementary Figure S8, doxorubicin alone increased caspase-3/7 activity to approximately two-fold at 20 µM, consistent with activation of caspase-dependent apoptotic signaling. In contrast, M3 alone did not increase caspase-3/7 activity. Notably, 2 µM M3 produced a caspase-3/7 signal of approximately 0.5-fold, despite producing a reduction in cell viability comparable to 20 µM doxorubicin under the same exposure conditions. Similarly, M3–doxorubicin combinations did not significantly increase caspase-3/7 activity beyond doxorubicin alone, despite producing enhanced reduction in cell viability. These findings suggest that the combined cytotoxic response is unlikely to be primarily driven by amplified caspase-dependent apoptosis. These findings provide mechanistic context for the Bliss-indicated interaction observed in the viability assay. The contrasting caspase-3/7 responses to doxorubicin and M3 suggest that the two agents may contribute to cytotoxicity through distinct or complementary mechanisms, rather than through a shared amplification of caspase-dependent apoptosis.

### 3.13. M3 Is Cytotoxic in Sensitive, Resistant, and Aggressive Cancer Cell Lines

M3 exhibited intrinsic low-micromolar cytotoxicity across lung, breast and ovarian cancer cell lines (Figure S9). This shows that this approach can be potentially used more widely on other cell lines too. The peptide reduced cell viability in both breast cancer models (MCF-7 and the aggressive triple-negative MDA-MB-231), small-cell lung cancer (H69) and its multidrug-resistant variant H69AR, as well as ovarian cancer (A2780 and the cisplatin-resistant A2780CIS). As a membrane-active peptide, the observed variation in IC_50_ values likely results from differences in membrane lipid composition, particularly cholesterol content. For example, the cholesterol-rich and rigid membranes of MDA-MB-231 are associated with higher IC_50_ values (4.20 ± 0.18 mM) compared to the more fluid membranes of MCF-7 (2.74 ± 0.25mM) [64]. This observation aligns with previous findings in HepG2 cells, where cholesterol depletion significantly increased melittin cytotoxicity, indicating that elevated cholesterol stabilizes membranes and reduced peptide-induced disruption [65,66]. The preserved broad-spectrum cytotoxicity of M3 in so many cell lines and particularly in H69AR and A2780CIS suggests that classical drug-efflux pathways are not likely to offer resistance against its powerful membrane-lytic action.

While the µM-range concentrations of M3 used in the combination treatment may not directly reflect clinically achievable exposure levels, these findings provide an initial proof-of-concept for M3-mediated enhancement of intracellular doxorubicin accumulation. Future optimization of the M3 scaffold, including increasing drug-binding affinity through conformational stabilization of the WXXW binding motif, may enable similar biological effects at lower concentrations. In addition, targeted delivery strategies, such as nanocarrier-based systems, may facilitate tumour-selective accumulation and improve the safety and pharmacokinetic profile of this approach, ultimately enhancing its translational potential.

## 4. Conclusions

A melittin peptide analogue was rationally engineered to achieve dual functionality, namely membrane activity with drug-binding capability. M3 preserves the critical α-helical structure required for membrane interaction and contains a defined drug-binding motif. Biophysical assays confirmed reversible binding of doxorubicin to M3 with moderate affinity. Molecular dynamics simulation in aqueous solution demonstrated that doxorubicin binding is initially anchored by the WXXW motif and subsequently transitions to a broader W12–W15–W19 (WXXWXXXW) aromatic interface. These multivalent interactions appear to enable M3 to transiently envelop doxorubicin, potentially masking efflux-recognition features and supporting a reversible, adaptive binding mode. Functionally, M3 increased intracellular calcein and doxorubicin accumulation in H69AR cells, indicating enhanced permeability and partial evasion of MRP1-mediated efflux. These uptake-enhancing effects were associated with synergistic cytotoxicity when M3 was combined with elevated doxorubicin concentrations after 48 h. Importantly, M3 maintained broad low-micromolar anticancer activity across six cancer cell lines, including resistant phenotypes, and demonstrated preferential toxicity toward H69AR cells compared to normal BEAS-2B cells. These findings highlight the potential of M3 as a selective, membrane-active, reversible drug-binding carrier.

## Figures and Tables

**Table 1:** Sequences and key features of melittin, Popkov A4, and the designed analogue.

Peptide	Sequence (N-C)	Key Features
Popkov A4	LWSPWYGGSW-NH_2_	Drug binding
Melittin	GIGAVLKVLTTGLPALISWIKRKRQQ-NH_2_	Membrane active
M3	GIGAVLKVLTLWSPWLISWIKRKRQQ-NH_2_	Hybrid

## Data Availability

The data presented in this study are available from the corresponding author upon reasonable request. The data are not publicly available as they form part of an ongoing research project.

## Supplementary Materials

The following supporting information can be downloaded at: https://www.mdpi.com/article/10.3390/pharmaceutics18070853/s1, Figure S1: Helical wheel projections of melittin and M3; Figure S2: Liposome leakage and circular dichroism analysis of melittin and M3; Figure S3: Time-dependent binding progression of M3–doxorubicin complex; Figure S4: Characterization of multidrug-resistant H69AR cell phenotype; Figure S5: Cytotoxicity profile of M3 in H69AR and BEAS-2B cells; Figure S6: Confocal analysis of doxorubicin uptake in H69AR cells; Figure S7: Cell viability of H69AR with M3, doxorubicin and combination; Figure S8: Effect of M3, doxorubicin, and their combination on caspase-3/7 activity in H69AR cells; Figure S9: Cytotoxicity of M3 across multiple cancer cell lines. Table S1: Bliss combination index (CI) for the combination of M3 and doxorubicin.

## Abbreviations

The following abbreviations are used in this manuscript:

ABC	ATP-binding cassette
ACPYPE	AnteChamber Python Parser interfacE
ANOVA	Analysis of variance
BEAS-2B	Bronchial epithelial cell line
BSA	Bovine serum albumin
CD	Circular dichroism
DCM	Dichloromethane
DMEM	Dulbecco’s Modified Eagle Medium
DMF	Dimethylformamide
DMSO	Dimethyl sulfoxide
DPBS	Dulbecco’s phosphate-buffered saline
EC_50_	Half maximal effective concentration
EDC	1-ethyl-3-(3-dimethylaminopropyl) carbodiimide
ESI-MS	Electrospray ionization mass spectrometry
FBS	Fetal bovine serum
GRAVY	Grand average of hydropathy
HBTU	O-(benzotriazole-1-yl)-N,N,N′,N′-tetramethyluronium hexafluorophosphate
HPLC	High-performance liquid chromatography
IC_50_	Half maximal inhibitory concentration
KD	Equilibrium dissociation constant
LINCS	Linear constraint solver
M3	Melittin analogue 3
MCF-7	Breast cancer cell line
MD	Molecular dynamics
MDA-MB-231	Triple-negative breast cancer cell line
MDR	Multidrug resistance
MM-GBSA	Molecular mechanics Generalized Born surface area
MM-PBSA	Molecular mechanics Poisson–Boltzmann surface area
MRP1	Multidrug resistance-associated protein 1
MST	Microscale thermophoresis
NHS	N-hydroxysuccinimide
NPT	Constant number of particles, pressure, and temperature
NVT	Constant number of particles, volume, and temperature
PBS	Phosphate-buffered saline
PDB	Protein Data Bank
Pgp	P-glycoprotein
PME	Particle mesh Ewald
Rg	Radius of gyration
RMSD	Root mean square deviation
RMSF	Root mean square fluctuation
RP-HPLC	Reverse-phase high-performance liquid chromatography
RU	Response unit
SD	Standard deviation
SPPS	Solid-phase peptide synthesis
TFA	Trifluoroacetic acid
TFE	Trifluoroethanol
TIS	Triisopropylsilane
