# Supplementary material for: Engineered Melittin Delivers a Drug-Loaded ‘Chemo-Sting’ to Overcome Efflux-Mediated Multidrug Resistance in Cancer Cells
Source: Pharmaceutics. 2026 Jul 14;18(7):853. doi: 10.3390/pharmaceutics18070853 (PMC13415283; doi:10.3390/pharmaceutics18070853)
Supplement: Supplementary file 1 [file pharmaceutics-18-00853-s001.zip › pharmaceutics-4382287-supplementary.pdf]

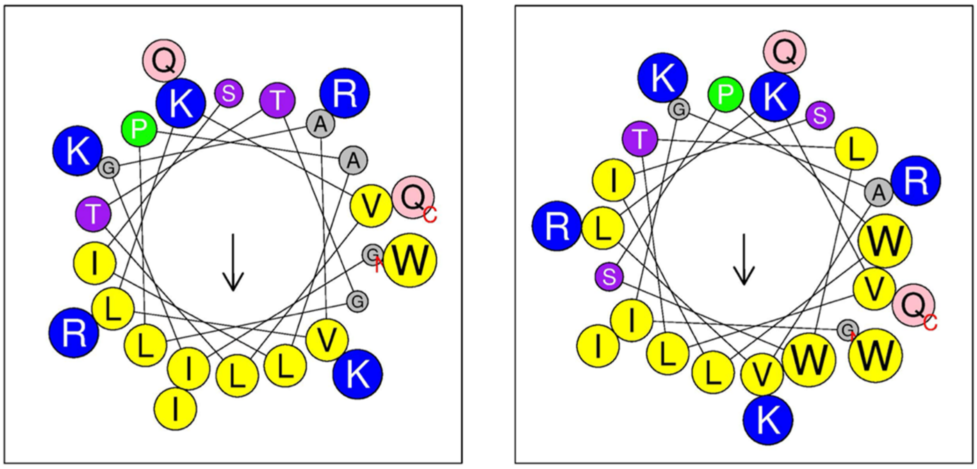

Figure S1: Helical wheel projections of melittin (left) and M3 (right) generated using HeliQuest. Hydrophobic residues are shown in yellow, basic residues in dark blue, polar uncharged residues in purple (serine, threonine) and pink (glutamine), glycine and alanine in grey, and proline in green. The arrow represents the hydrophobic moment, indicating the direction and magnitude of amphipathicity. Melittin displays a clear hydrophobic face, whereas M3 shows a more dispersed hydrophobic pattern and a shorter hydrophobic moment arrow, reflecting reduced amphipathicity.

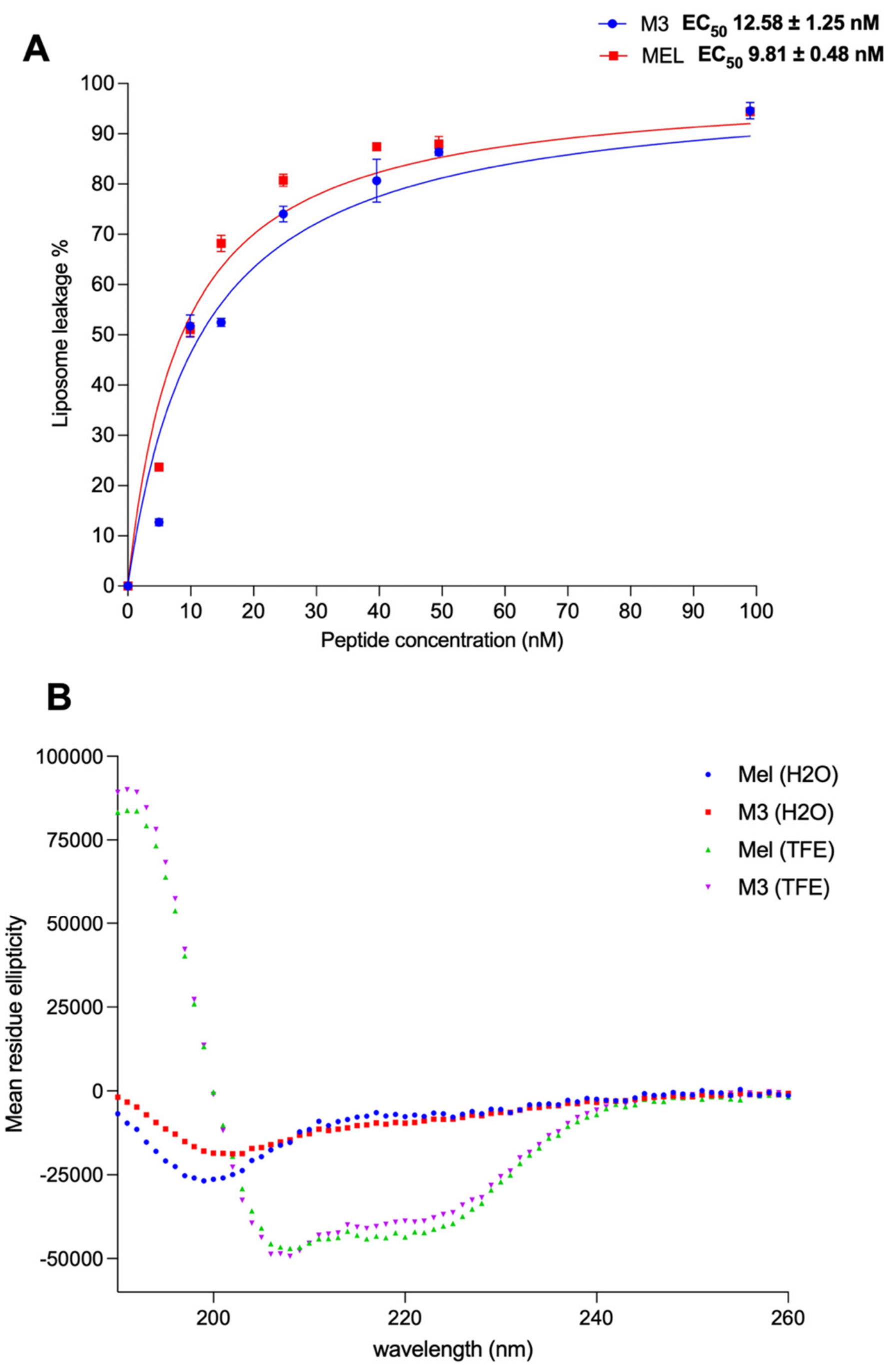

Figure S2: Comparative membrane disruption and helical transition of melittin and M3. (a) Liposome leakage induced by melittin and M3 across increasing peptide concentrations (nM). Leakage was quantified as percent calcein release using liposomes containing 75 µg of total lipid per test, with Triton X-100 used as the positive control to define 100% leakage and enable calculation of EC_{50} values. (b) Circular dichroism spectra of melittin and M3 recorded in water and in 50% TFE, showing the coil-to-helix transition characteristic of peptide folding under membrane-mimetic conditions.

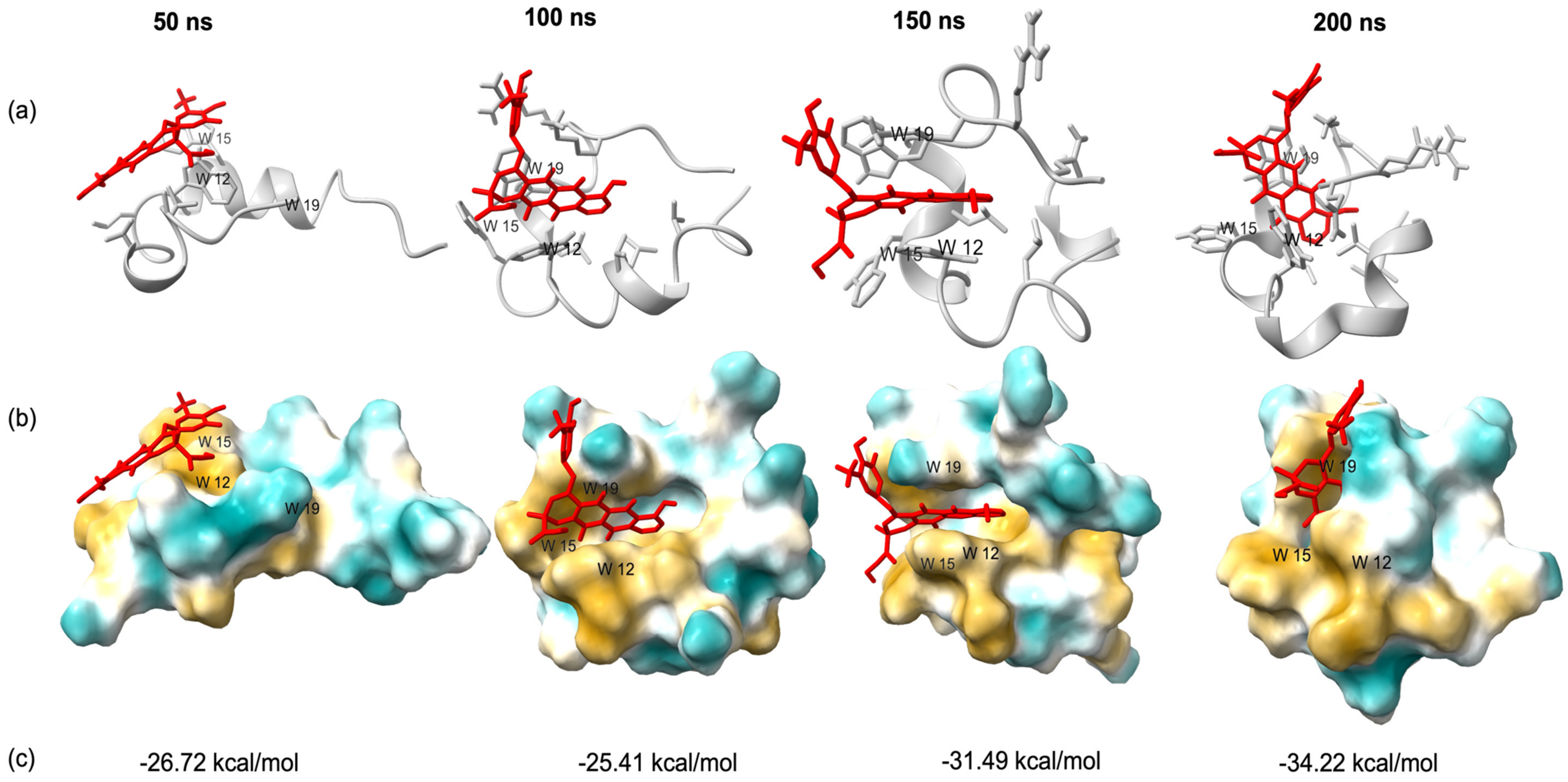

Figure S3: Binding progress. (a) Dynamic profile of melittin analogue (grey) and doxorubicin (red) complex every 50 ns throughout simulation start with 50 ns snapshot from the very left. (b) hydrophobic peptide surface representation showing progress of doxorubicin buried into the peptide. Peptide surface was coloured from brown (hydrophobic) region to turquoise (hydrophilic) region. (c) binding energy at designated time frame.

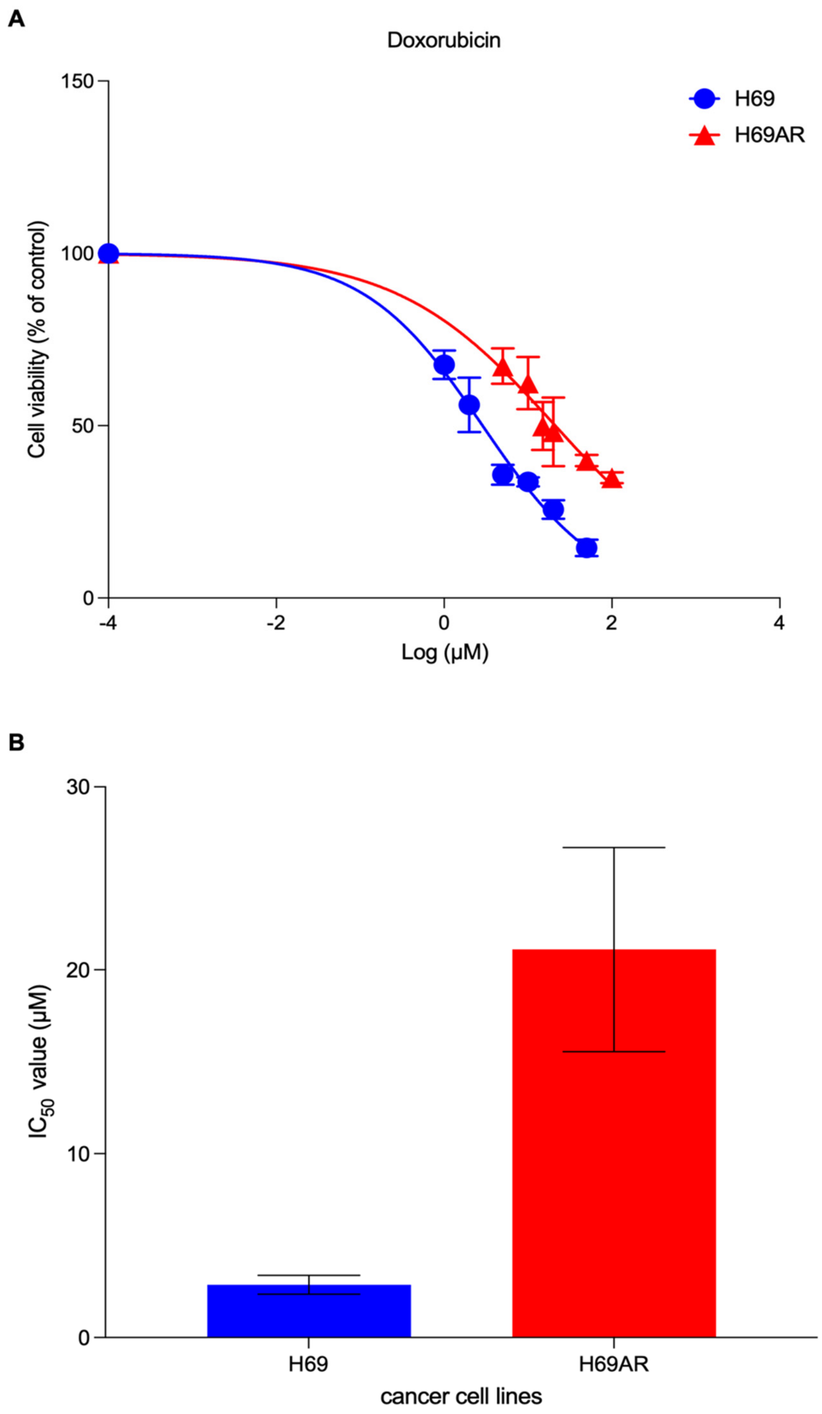

Figure S4: Characterisation of MDR phenotype in H69AR cells. (a) Dose–response curves showing doxorubicin-induced cytotoxicity in H69 and H69AR cells, with viability measured after 24 hours and plotted against log-transformed doxorubicin concentration. (b) Comparison of doxorubicin IC_{50} values between H69 and H69AR, confirming elevated resistance in H69AR cells

Selective cytotoxicity of M3 in H69AR in comparison to normal cell BEAS-2B

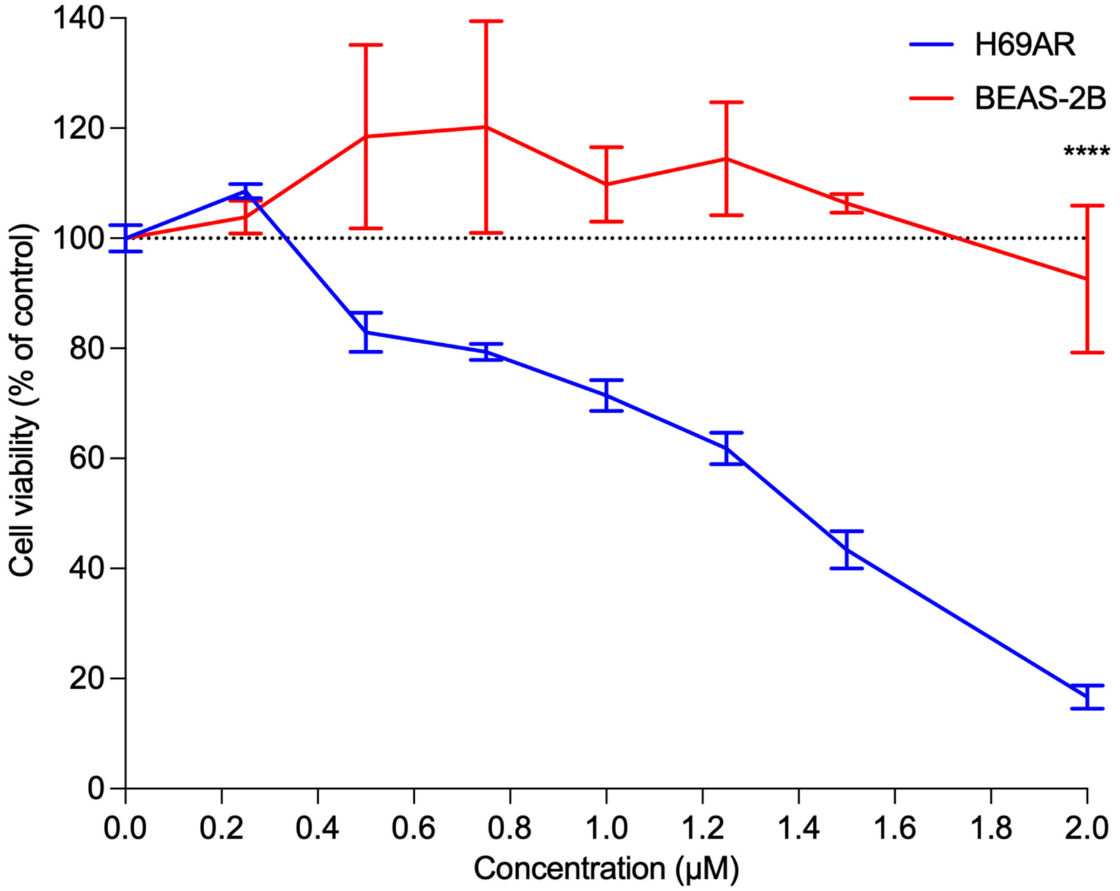

Figure S5: Concentration-response analysis of M3 in H69AR and BEAS-2B cells. Cell viability was measured after 24 hours of treatment with increasing concentrations of M3 using the CellTiter-Blue assay. Data represent (mean ± SD, n = 3).

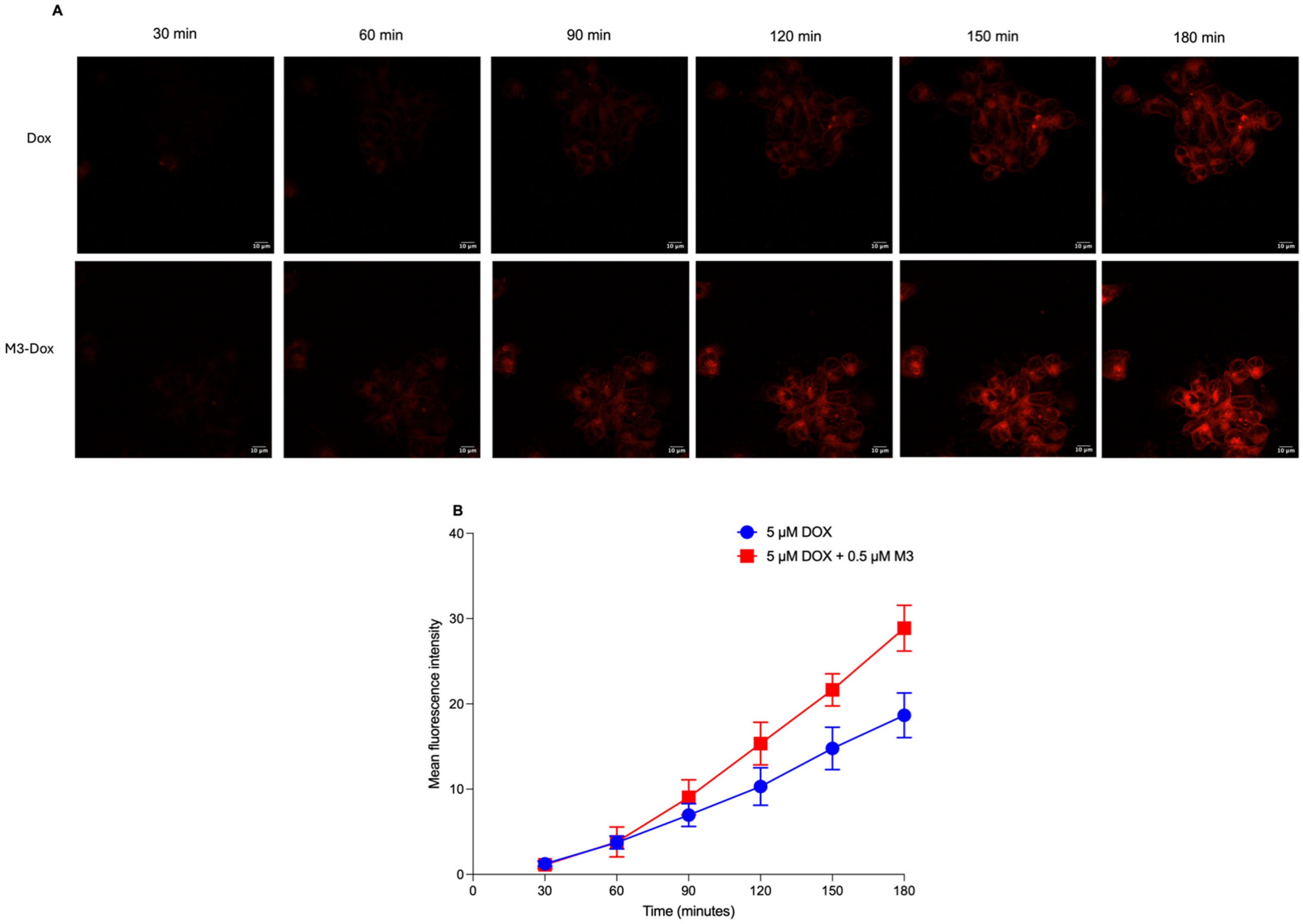

Figure S6: Confocal analysis of doxorubicin uptake in H69AR cells. (A) Representative live-cell confocal images captured at 30-minute intervals over a 3-hour period comparing doxorubicin alone (upper panel) with the M3–doxorubicin combination (lower panel). Scale bar: 10 µm. (B) Mean fluorescence intensity profile of intracellular doxorubicin over time (mean ± SD, n = 3)

Table S1: Bliss combination index (CI) for M3 and doxorubicin co-treatments

Doxorubicin (μM)	1 μM		2 μM	
	**24 h**<br>**CI ± SD**	**48 h**<br>**CI ± SD**	**24 h**<br>**CI ± SD**	**48 h**<br>**CI ± SD**
**5**	1.16 ± 0.16<br>Antagonistic	1.00 ± 0.08<br>Additive	1.17 ± 0.02<br>Antagonistic	1.05 ± 0.10<br>Additive
**10**	1.00 ± 0.08<br>Additive	0.93 ± 0.07<br>Synergistic	1.12 ± 0.04<br>Antagonistic	0.96 ± 0.09<br>Synergistic
**15**	1.01 ± 0.14<br>Additive	0.91 ± 0.08<br>Synergistic	1.03 ± 0.02<br>Additive	0.87 ± 0.002<br>Synergistic
**20**	0.95 ± 0.12<br>Synergistic	0.87 ± 0.05<br>Synergistic	1.00 ± 0.02<br>Additive	0.90 ± 0.02<br>Synergistic

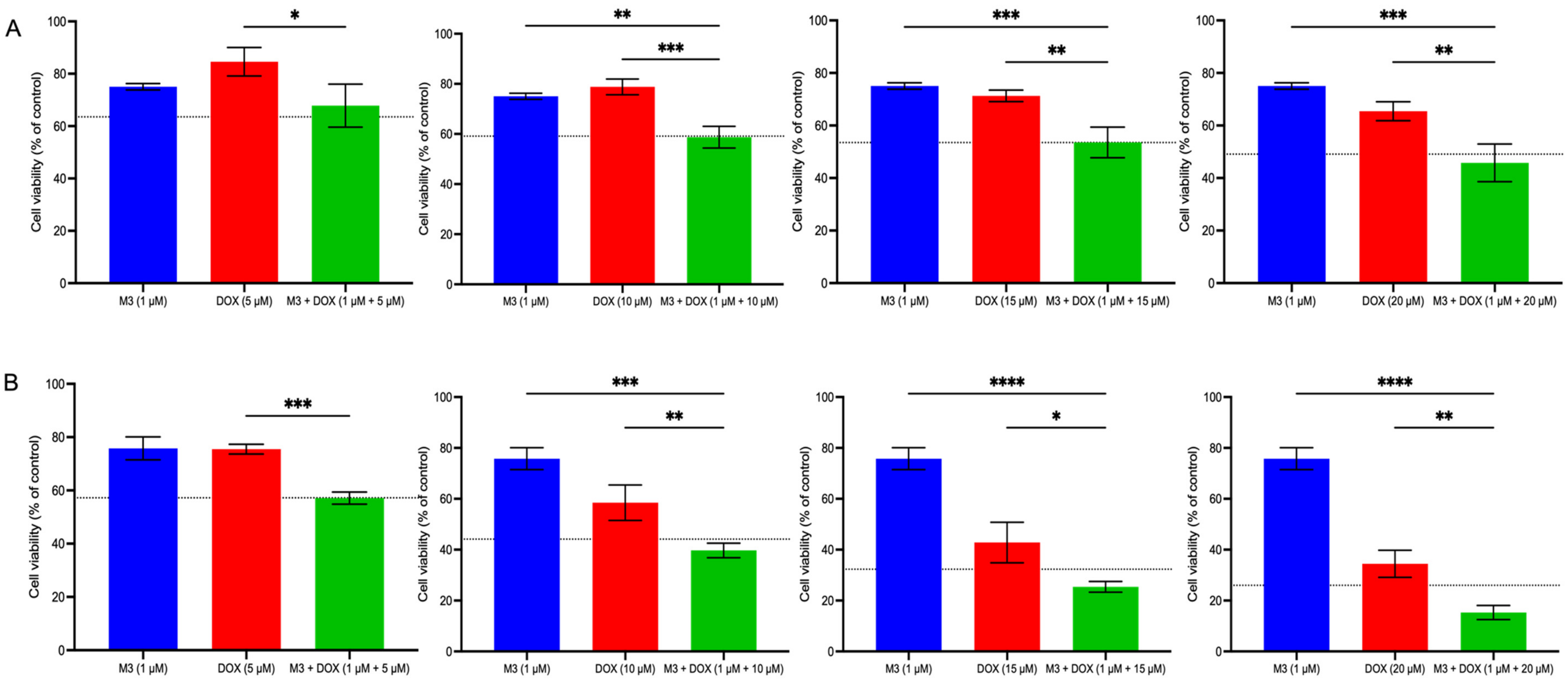

Figure S7a: Cell viability of H69AR cells treated with 1 µM M3 for 24 hours (panel A) and 48 hours (panel B) in combination with increasing doxorubicin (5 mM, 10 mM, 15 mM, and 20 mM). Data are presented as mean ± SD (n=3). Statistical analysis was performed using one-way ANOVA followed by Tukey's multiple comparison test to evaluate differences between treatment groups. Significance levels are denoted as ns (not significant, $p \geq 0.05$), *$p < 0.05$, **$p < 0.01$, ***$p < 0.001$, and ****$p < 0.0001$. A dotted horizontal line represents the Bliss-expected additive effect; bars below the line indicate synergy, while bars above the line indicate antagonism.

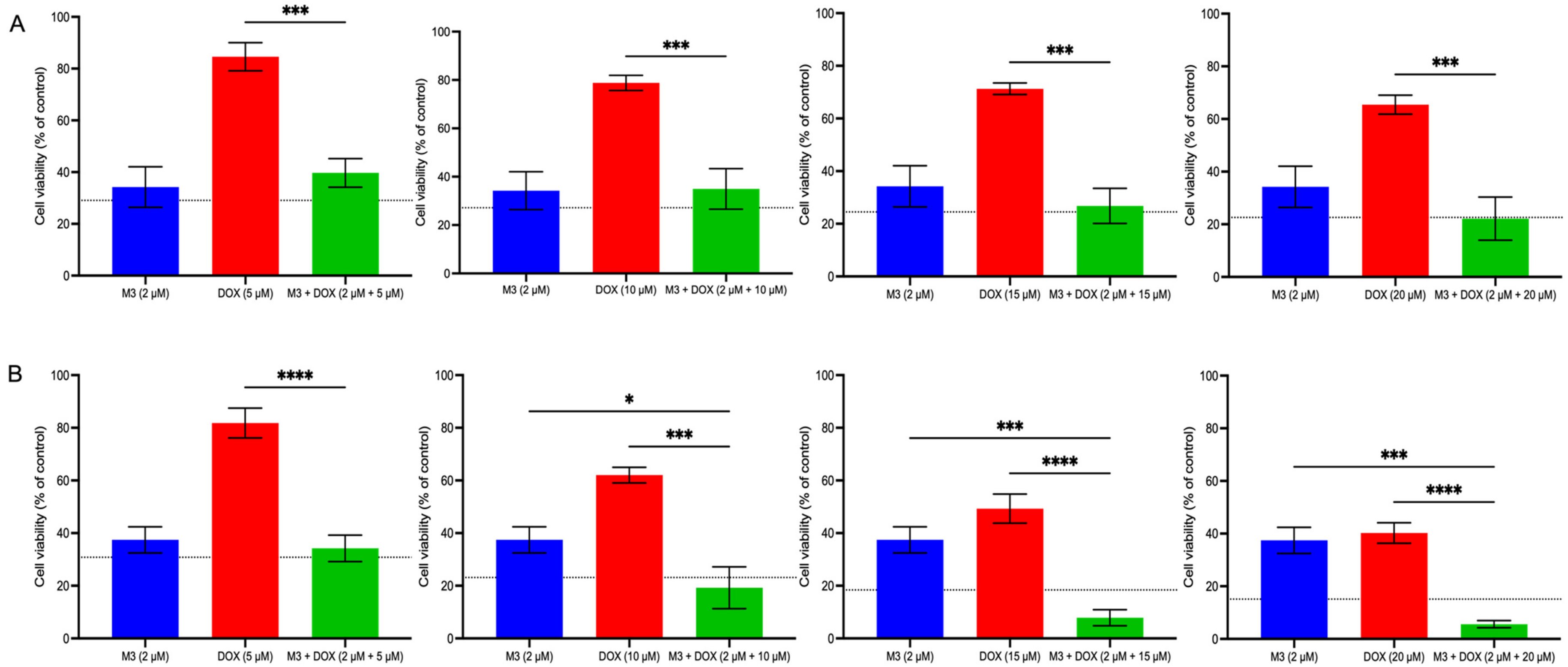

Figure S7b: Cell viability of H69AR cells treated with 2 µM M3 for 24 hours (panel A) and 48 hours (panel B) in combination with increasing doxorubicin (5 µM, 10 µM, 15 µM, and 20 µM). Data are presented as mean ± SD (n=3). Statistical analysis was performed using one-way ANOVA followed by Tukey's multiple comparison test to evaluate differences between treatment groups. Significance levels are denoted as ns (not significant, $p \geq 0.05$), *$p < 0.05$, **$p < 0.01$, ***$p < 0.001$, and ****$p < 0.0001$. A dotted horizontal line represents the Bliss-expected additive effect; bars below the line indicate synergy, while bars above the line indicate antagonism.

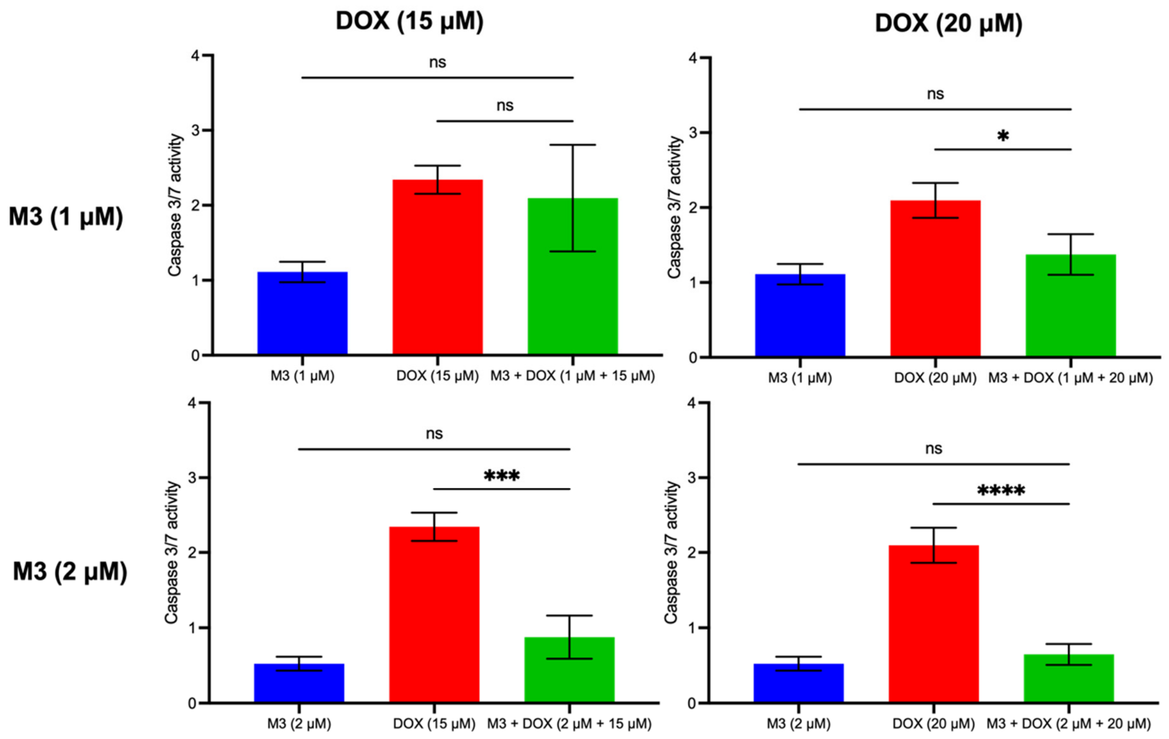

Figure S8: Effect of M3, doxorubicin, and their combination on caspase-3/7 activity in H69AR cells. Caspase-3/7 activity in H69AR cells following 48-hour treatment with M3, doxorubicin (DOX) and their combination. Cells were seeded at 10,000 cells per well in 96-well plates and treated with doxorubicin (15 µM or 20 µM), M3 (1 µM or 2 µM), or their combination under the same conditions. Caspase-3/7 activity was measured using a luminescence-based assay according to the manufacturer's instructions. Luminescence was recorded using a microplate reader and normalised to untreated control cells. Data are presented as mean ± SD from three independent experiments (n = 3). Statistical analysis was performed using one-way ANOVA followed by Tukey's multiple comparisons test. Significance levels are denoted as ns (not significant, $p \geq 0.05$), *$p < 0.05$, **$p < 0.01$, ***$p < 0.001$, and ****$p < 0.0001$.

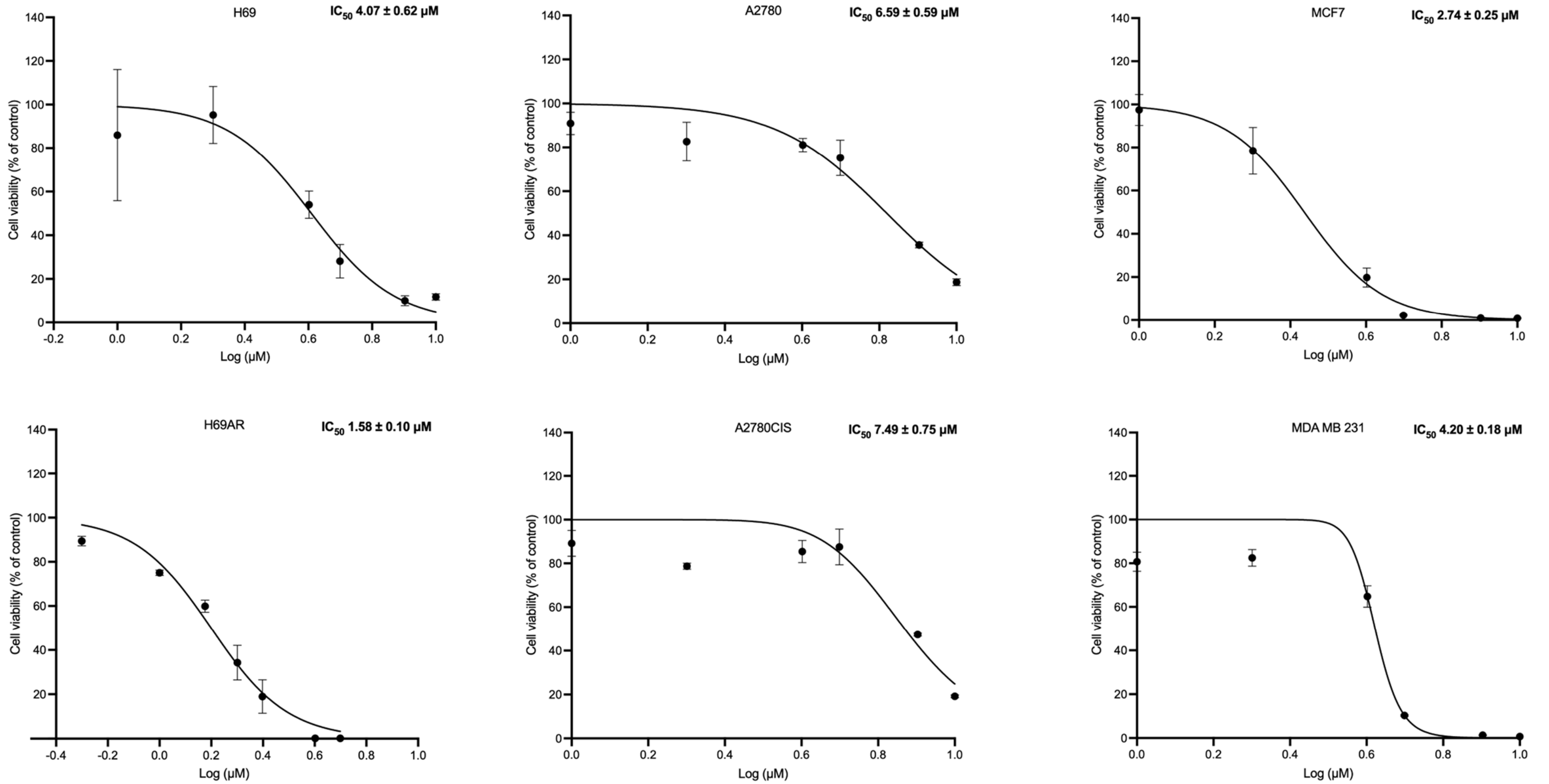

Figure S9: M3 peptide exhibits sub-10 μM cytotoxicity across lung, breast, and ovarian cancer cell lines, including sensitive, resistant and aggressive phenotype. The cell viability was assessed using CellTiter-blue assay and expressed as percentage relative to untreated control cells. Data represent mean ± standard deviation (n=3)
